# The genome sequence of the Antarctic bullhead notothen reveals evolutionary adaptations to a cold environment

**DOI:** 10.1186/s13059-014-0468-1

**Published:** 2014-09-25

**Authors:** Seung Chul Shin, Do Hwan Ahn, Su Jin Kim, Chul Woo Pyo, Hyoungseok Lee, Mi-Kyeong Kim, Jungeun Lee, Jong Eun Lee, H William Detrich, John H Postlethwait, David Edwards, Sung Gu Lee, Jun Hyuck Lee, Hyun Park

**Affiliations:** Division of Polar Life Sciences, Korea Polar Research Institute, Yeonsu-gu, Incheon 406-840 South Korea; Polar Sciences, University of Science & Technology, Yuseong-gu, Daejeon 305-333 South Korea; Division of Biotechnology, Korea University, Sungbuk-gu, Seoul 406-840 South Korea; Fred Hutchinson Cancer Research Center, 1100 Fairview Avenue North, D4-100, Seattle, WA 98109-1024 USA; DNA Link, Inc, Songpa-gu, Seoul 138-736 South Korea; Department of Marine and Environmental Sciences, Marine Science Center, Northeastern University, Nahant, MA 01908 USA; Department of Biology, University of Oregon, Eugene, OR 97403 USA; Australian Centre for Plant Functional Genomic, School of Agriculture and Food Sciences, University of Queensland, St Lucia, QLD Australia; School of Plant Biology, University of Western Australia, Crawley, WA 6009 Australia

## Abstract

**Background:**

Antarctic fish have adapted to the freezing waters of the Southern Ocean. Representative adaptations to this harsh environment include a constitutive heat shock response and the evolution of an antifreeze protein in the blood. Despite their adaptations to the cold, genome-wide studies have not yet been performed on these fish due to the lack of a sequenced genome. *Notothenia coriiceps*, the Antarctic bullhead notothen, is an endemic teleost fish with a circumpolar distribution and makes a good model to understand the genomic adaptations to constant sub-zero temperatures.

**Results:**

We provide the draft genome sequence and annotation for *N. coriiceps*. Comparative genome-wide analysis with other fish genomes shows that mitochondrial proteins and hemoglobin evolved rapidly. Transcriptome analysis of thermal stress responses find alternative response mechanisms for evolution strategies in a cold environment. Loss of the phosphorylation-dependent sumoylation motif in heat shock factor 1 suggests that the heat shock response evolved into a simple and rapid phosphorylation-independent regulatory mechanism. Rapidly evolved hemoglobin and the induction of a heat shock response in the blood may support the efficient supply of oxygen to cold-adapted mitochondria.

**Conclusions:**

Our data and analysis suggest that evolutionary strategies in efficient aerobic cellular respiration are controlled by hemoglobin and mitochondrial proteins, which may be important for the adaptation of Antarctic fish to their environment. The use of genome data from the Antarctic endemic fish provides an invaluable resource providing evidence of evolutionary adaptation and can be applied to other studies of Antarctic fish.

**Electronic supplementary material:**

The online version of this article (doi:10.1186/s13059-014-0468-1) contains supplementary material, which is available to authorized users.

## Background

Antarctic fish have experienced extraordinary evolutionary episodes since the cooling of the Southern Ocean to the freezing point of seawater (-1.9°C) about 34 million years ago after the opening of the Drake passage and the establishment of the Antarctic Circumpolar current, which led to thermal isolation and widespread glaciation of Antarctica [1,2]. Particularly in this environment, adaptations occurred including an antifreeze glycoprotein gene that evolved from a duplicated trypsinogen gene [3-5], cold-efficient microtubule assembly [6,7], loss of an inducible heat shock response [8-10], and changes in membrane fluidity [11]. The Channichthyidae (white-blooded icefish) clade of Notothenioids even lost functional hemoglobin, myoglobin, and the ability to make red blood cells [2,12,13]. The history of these evolutionary episodes can likely be decoded from explorations of the genomes of Antarctic fish and their compensatory adaptations to their near-freezing environment.

Waters of the Antarctic continental shelf and upper slope contain 222 species of fish from 19 families. The Notothenioids, a perciform group, account for 45.5% of the species [14]. In the high latitude (71S-78S) embayments of the Ross and Weddell Seas, Notothenioids dominate Antarctic fish fauna and represent 77% of the species diversity. It would be 92% of the number of individuals, and 91% of the biomass [15]. Ninety-seven percent of Antarctic Notothenioid fish are endemic [16]. *Notothenia coriiceps* (Richardson, 1844) is one of the major Antarctic fish for studies of adaptation in the Southern Ocean [3,17-19]. *N. coriiceps* is highly abundant in near-shore Antarctic waters and may have a circumantarctic distribution [20]. Here we discuss the sequencing and analysis of the genome of the Antarctic bullhead notothen, *N. coriiceps*, and report transcriptome analysis from RNA-seq experiments conducted to explore temperature challenges involved in cold-adapted evolution. We sequenced the genome of an Antarctic bullhead notothen, *N. coriiceps*, applying a whole genome shotgun approach to a total of 84.5× coverage for its estimated genome size of 637 Mb to understand these evolutionary mechanisms. This report illuminates evolutionary trajectory of some major life-history traits of these Antarctic fish, provides important clues for ecological and population studies designed to address issues of Antarctic biota, and contributes a reference genome for use in future comparative studies of Antarctic adaptations.

## Results

### Sequence and assembly

We sequenced genomic DNA extracted from a single *Notothenia coriiceps* collected at northern Antarctic Peninsula. We used three sequencing platforms: Illumina HiSeq2000, GS-FLX, and Pacbio *RS* with coverage of 78.6×, 2.0×, and 3.9×, respectively. Initial hybrid assemblies were performed using the Celera Assembler with Illumina short reads and 454 reads [21] (Additional file 1: Tables S1 and S2). A total of 25,794 assembly gaps were filled with Illumina reads and error-corrected continuous long reads (CLR) generated from Pacbio *RS* [22,23] (Additional file 1: Table S3 and Additional file 2: Figure S1). A total of 18,400 gaps were filled with Illumina reads using Gapfiller (Ver. 1.9) and 7,394 gaps were closed with CLR reads using PBjelly (Ver. 12.9.14). The final assembly consisted of 38,062 scaffolds that comprised 100,606 contigs spanning 637 Mb with remaining unclosed gaps of roughly 13.1 Mb (2.1% of the total scaffold sequence). To validate the final assembly accuracy of the scaffolds, we sequenced and assembled bacterial artificial chromosomes (BACs) clones using GS-FLX. Six sequenced BAC clones were aligned to the scaffolds, and 99% of the total BAC clones were identical to the assembled scaffolds (Additional file 2: Figure S2). The final assembly had an N50 contig size of 11.6 Kb and an N50 scaffold size of 219 Kb, and the largest scaffold was 28 Mb (Table 1).

**Table 1:** **Global statistics of the** ***N. coriiceps*** **genome assembly**

**Sequencing platform**	**Insert size**		**Total data (Mb)**	**Sequence coverage (×)**
Illumina paired-end	150, 300, 350, 500, 600 bp		47,163	78.6
GS-FLX Mate-pair	Single, 3, 8, 20 kb		1,185	2.0
PacBioRS	Continuous Long Read		2,318	3.9
**Assembly results**	**Number**	**N50 (kb)** ^**a**^	**ML (kb)** ^**b**^	**Size (Mb)**
Contig	100,606	11.6	226.8	622
Scaffold	38,062	219.1	28,796.7	637
**Annotation**	**Number**		**Total length (kb)**	**Percentage of genome**
Genes	32,260		47,712	7.5
Repeats			115,561	18.15

^a^Minimum sequence length in which half of the assembled bases were found.

^b^Maximum length.

### Genome annotation

For gene prediction, we used 36 Gb of RNA sequencing data from seven tissues (brain, skin, egg, kidney, muscle, stomach, and blood) and 300 Mb error-corrected CLR from three tissues (egg, skin, and muscle) (Additional file 1: Tables S4 and S5), and the MAKER annotation pipeline approach using both evidence-based and *ab initio* methods [24] resulting in a final gene set of 32,260 protein-coding genes. A total of 29,045 of these protein-coding genes were assigned preliminary functions with BLASTp, and we could assign Gene Ontology (GO) terms to 19,556 (60.57%) predicted genes based on BLASTp results and InterproScan, encompassing biological processes (14,602 (45.22%)), cellular components (12,511 (38.75%)), and molecular functions (15,972 (49.47%)) (Additional file 2: Figure S3). Enzyme commission (EC) was obtained for 3,465 proteins (Additional file 1: Table S6 and S7). Annotated genes contained an average of 6.65 exons, with an average mRNA length of 1,478 bp and CDS length of 1,063 bp. The *de novo* repeat prediction showed that repeat sequences accounted for 18.15% of the assembled *N. coriiceps* genome (Additional file 1: Table S8) and 529 tRNA were also predicted (Additional file 1: Table S9).

### The evolution of gene families in *N. coriiceps*

**Figure 1:**
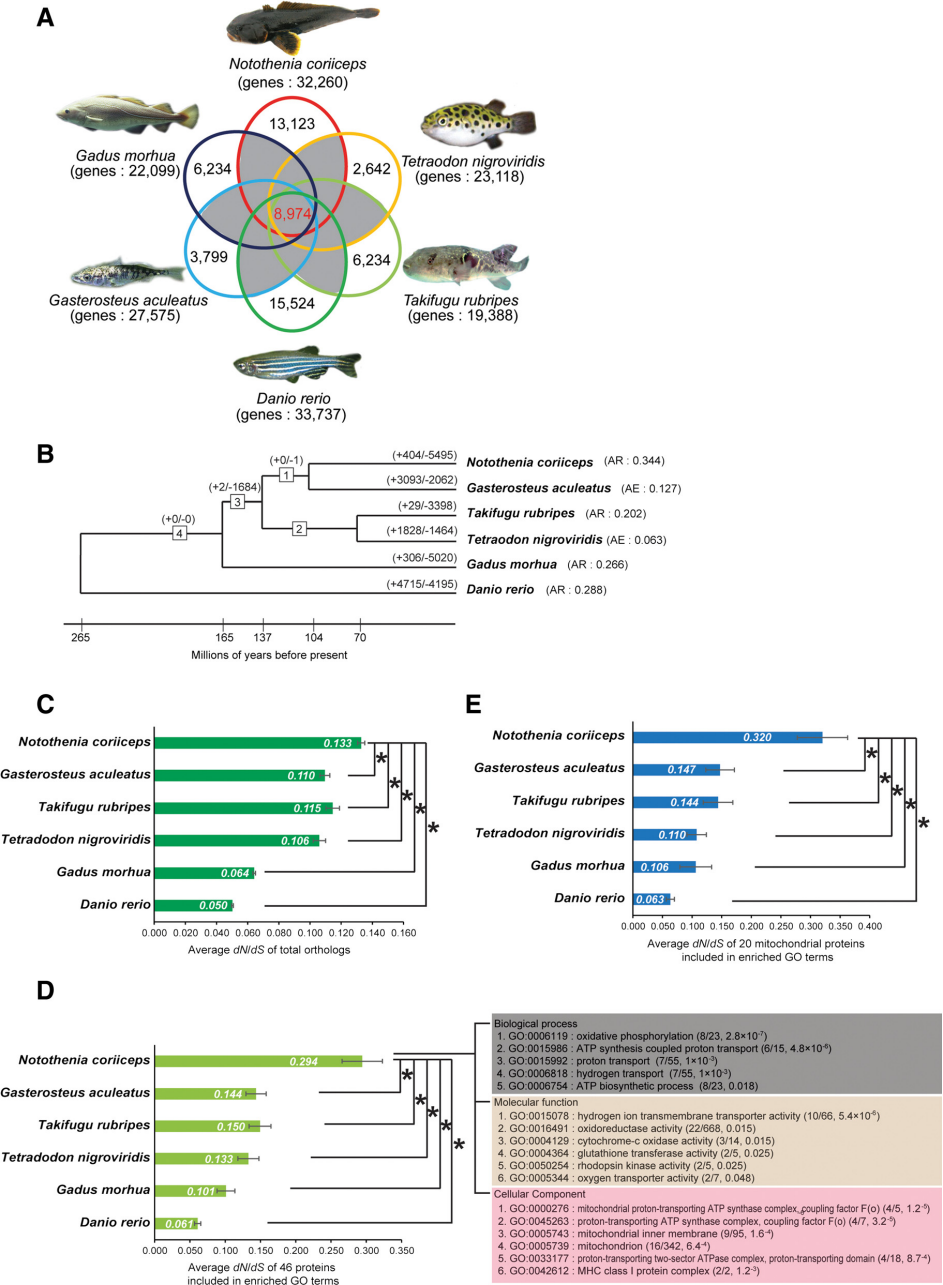
**Genome-wide analysis of protein-coding genes in** ***N. coriiceps.*** **(A)** Venn diagram displaying the overlap in gene families in six fish species. A total of 18,131 gene families that are included in gray background were used to analyze gain and loss of gene in six fish. **(B)** Lineage-specific genes expansion and contraction among six fish. The numbers in boxes are identifiers for internal branches of the phylogeny. Numbers on each branch denote the number of gene gains (+)/losses (-). AE and AR denote average expansion family (mean number of genes gained) and average reduction family (mean number of genes lost), respectively. **(C)** The average dN/dS of 5,039 orthologs were determined. Bar charts show the average dN/dS values for six fish species. Data were analyzed using an analysis of variance (ANOVA) followed by Bonferroni post hoc test; values represent mean ± SEM (*P <0.001). **(D)** In a GO enrichment test among the rapid-evolving genes with dN in the top 10%, 17 GO terms including 46 genes were significantly enriched in *N. coriiceps*. The average dN/dS of 46 proteins included in enriched GO terms. Bar charts show the average dN/dS values for six fish species. Data were analyzed using an analysis of variance (ANOVA) followed by Bonferroni post hoc tests; values represent mean ± SEM (*P <0.001). **(E)** The average dN/dS ratio of 20 mitochondrial proteins included in enriched GO terms. Bar charts show the average dN/dS values for six fish species. Data were analyzed using an analysis of variance (ANOVA) followed by Bonferroni post hoc test; values represent mean ± SEM (*P <0.001).

Gene families are groups of homologous genes that possess highly identical structures and similar functions. These families vary in gain or loss of genes, making the size different among gene families through evolution [25-27]. To identify the evolution of *N. coriiceps* gene families, we investigated the size differences between 18,131 gene families with at least two genes across six fish of interest (*Danio rerio*, *Gasterosteus aculeatus*, *Takifugu rubripes*, *Tetraodon nigroviridis*, *Gadus morhua*, and *N. coriiceps*) (Figure 1A and B). We were able to identify the largest number of gene family contractions (5,495 gene families) and the average reduction (0.344), which means number of gene lost per family, in the *N. coriiceps* lineage. The lineage *D. rerio* has the largest number of gene family expansions (4,715) among these six fish. In the likelihood approach to studying gene family evolution, gene families evolving at significant rates of gain and loss than the genome wide average could exhibit higher expansions or contractions [27]. From the 18,131 gene families, 82 showed significant expansions or contractions among six fishes at *P* <0.0001 [26-29]. At this significance level, only one family is expected by chance (a false discovery rate = 0.02%); finally we identified 32 families showing significantly difference in *N. coriiceps* lineage. However, we were not able to identify significant expansions in *N. coriiceps*, but only significant contractions occurred in 32 gene families (Additional file 3).

To investigate selective pressures on protein-coding genes of *N. coriiceps*, we determined the dN/dS ratio (the ratio of the rate of non-synonymous substitutions to the rate of synonymous substitutions) of 8,974 orthologs in six fish (Figure 1A, Additional file 1: Table S10 and Additional file 2: Figure S4). Orthologs showing poor alignment were removed, and those with high synonymous substitution rates (over 3) and excessive transition/transversion ratios (over 10) were also removed. Finally, the dN/dS of 5,039 orthologs were determined. The average dN/dS ratio of *N. coriiceps* (0.133) was significantly higher than that of the other five fish (range: 0.050 to 0.115) (Figure 1C). We interpret this comparison to indicate a high level of selective pressure caused by the harsh Antarctic environment. To determine which functional gene categories evolved most rapidly, we selected 505 rapidly evolving genes with dN, as an indicator to categorize fast and slow evolving orthologs, in the top 10% of 5,039 genes in *N. coriiceps*, then analyzed them for statistically over-represented genes in the Gene Ontology [30-32] (Additional file 1: Table S11 and Additional file 4). Seventeen GO terms (included 46 genes) were significant in GO enrichment analysis using selected 505 rapidly evolving genes (Figure 1D and Additional file 1: Table S12). The average dN/dS ratio (0.294) of 46 genes in *N. coriiceps* was statistically higher than the orthologs of these 46 genes in the other five fish (approximately 0.061 to 0.150). It is noteworthy that 13 GO terms among enriched 17 GO terms were associated with mitochondria (Additional file 1: Table S12). We focused on 20 mitochondrial protein-coding genes that were enriched in 13 GO terms (Additional file 1: Table S12). Results showed that the average dN/dS ratio (0.320) for this set of 20 *N. coriiceps* mitochondrial genes was also statistically higher than other fish mitochondrial genes (approximately 0.063 0.147) (Figure 1E). Our observation that most GO terms linked to rapidly evolved mitochondrial genes in *N. coriiceps* might be functions correlated with high thermal sensitivity in Antarctic notothenioids [33-35]. Other GO terms significantly enriched among rapidly evolving genes were glutathione transferase activity (GO:0004364), rhodopsin kinase activity (GO:0050254), and oxygen transporter activity (GO:0005344) in molecular function, and MHC class I protein complex (GO:0042612) in cellular component. Oxygen transporter activity (GO:0005344) among significant enriched GO terms including alpha and beta globins might be also associated with mitochondria through supplying oxygen for their oxidative phosphorylation.

We also investigated whether the rapidly evolving genes as defined by dN are specific to the *N. coriiceps* lineage and whether the results have been due to positive selection or relaxation of selection pressure. We used a branch-specific model, and identified that the dN/dS of 117 genes were significantly different from the rest of the phylogenetic tree of six fish. Seventy-two genes (including 10 mitochondrial genes) among 117 genes (including 14 mitochondrial genes) were under significant positive selection (Additional file 1: Table S11), and also the oxidative phosphorylation (GO:0006119) of GO term was statistically represented in the GO enrichment test for genes under positive selection.

### Heat shock factor in *N. coriiceps*

**Figure 2:**
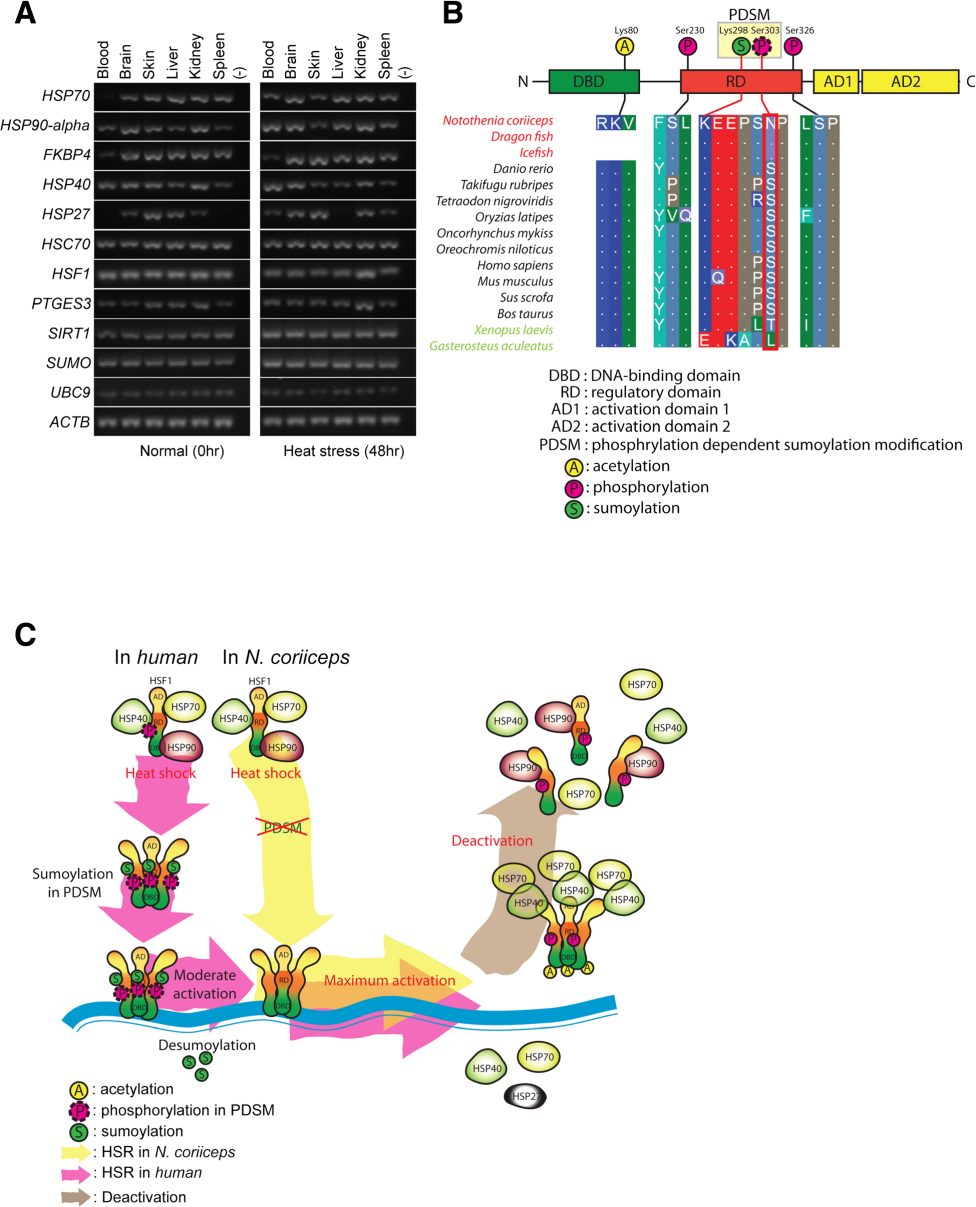
**Heat shock response in** ***N. coriiceps*** **. (A)** Tissue specific expression of HSR-related genes in various tissues. Multi-chaperone complexes of HSP90 (*FKBP* and *PTGES*), *SIRT1* (NAD-dependent decetylase sirtuin-1; *SIRT1* is negatively related to the acetylation of *HSF1* in humans), *SUMO*, *HSP90-alpha*, and *UBC9* were expressed in blood and other tissues. The SUMO-cconjugation enzyme ubiquitin carrier 9 (*UBC9*) discriminates between the phosphorylated and non-phosphorylated PDSM of HSF1. **(B)** HSF1 and its post-translational modification sites in *N. coriiceps*. Serine corresponding to ser303 of human *HSF1* was substituted with asparagine. This site is responsible for sumoylation of lys298 of PDSM in human hsf1. In *Xenopus* sp. and *G. aculeatus*, other amino acids were identified at the site corresponding to ser303 of human HSF1. All sequenced mammals and most fish have serine at this site. This substitution of serine to asparagine was also identified in icefish and Dragon fish in the Antarctic Ocean. **(C)** Components responsible for the HSR in *N. coriiceps* and its regulation.

Although the heat shock response (HSR), a defense mechanism against thermal stress, is inducible in most animals, Antarctic notothenioid fish have been reported to lack an inducible HSR [12,31]. In contrast, HSR proteins in Antarctic fish are constitutively expressed, presumably to mitigate cold denaturation of proteins [10,36-39]. To identify whether the loss of gene affects the constitutive HSR expression with the cold denaturation of proteins [40], we investigated the genes related to the regulation of the HSR in the *N. coriiceps*, and identify that HSR-related genes were well-conserved in their draft genome and their gene expressions were also identified by reverse transcriptase PCR (rt-PCR) in normal condition or in stress condition (Figure 2A). In vertebrate HSR, the heat shock factor 1 (*HSF1*) gene is known as the master regulator. Therefore, we investigate whether the functional domain of *HSF1* was well conserved. As a result, *HSF1* lost its phosphorylation-dependent sumoylation motif (PDSM), which is essential for repressing its transactivation capacity (Figure 2B and Additional file 2: Figure S5). Phosphorylation of the serine residue in the PDSM (KxExxSP) is a prerequisite for conjugation of a small ubiquitin-related modifier peptide (SUMO) to a single lysine residue in *HSF1*. When maximal *HSF1* activity is required, desumoylating enzymes remove this modification from *HSF1* [41-44] (Figure 2B and C). We found that the serine residue of PDSM in *HSF1* was substituted with asparagine in *N. coriiceps* and in other Antarctic fish as well including the icefish *Chaenocephalus aceratus* and the dragonfish *Parachaenichthys charcoti*) (Additional file 2: Figure S5). In response to thermal stress, the DNA-binding and transactivation capacity of *HSF1* are coordinately regulated through multiple post-translational modifications (PTMs), protein-protein interactions and subcellular localization [41]. Loss of this sumoylation site of PDSM (Ser303 to Asn303) would allow for maximal activation in response to heat shock stress with simple methods in the genomic context of *N. coriiceps* (Figure 2C) [41,43].

### Heat shock response in *N. coriiceps*

**Figure 3:**
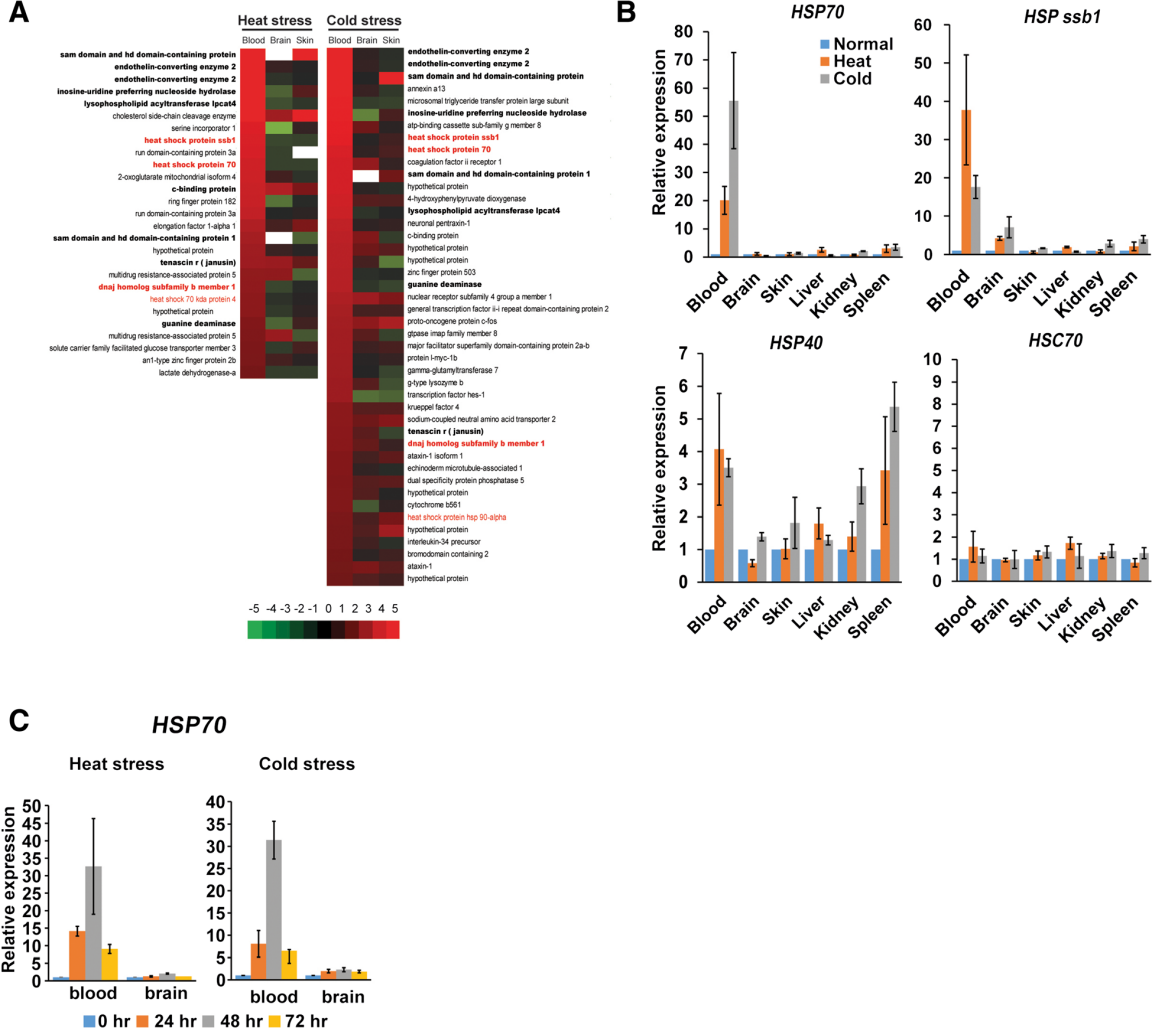
**RNA-Seq analysis of** ***N. coriiceps*** **blood, brain, and skin tissues under cold and heat stress. (A)** Genes significantly upregulated in response to heat stress or cold stress in whole blood (>two-fold). Bold characters represent genes shared between both stresses. Red characters represent genes related to the HSR. Expression value is on a log scale. Gene order was based on expression levels in whole blood. **(B)** qPCR results of *HSP70*, *HSP40*, *Heat shock protein ssb1*, and heat shock cognate protein 70 gene (*HSC70*). Beta-actin was used as a control. **(C)** Time course of *HSP70* gene expression. The expression level of the *HSP70* gene increased with increasing time exposure to heat or cold stress up to 48 h and decreased after 24 h of stress recovery.

To address the question of response to heat shock, raised by our finding of the loss of the sumoylation site in the *N. coriiceps* HSP1 protein, we investigated the HSR in these fish. *N. coriiceps* is a stenothermal fish that can survive only within the range of -2.5°C to 6.0°C [45]. When we exposed *N. coriiceps* to heat shock (4°C) for 48 h, 32 genes (including *HSP70*, *HSP40*, and *Heat shock protein ssb1*) were significantly upregulated more than two-fold based on the RNA-seq analyses (Figure 3A, Additional file 1: Tables S13 and S14). We confirmed that HSR-related genes were upregulated in the whole blood sample using qPCR (Figure 3B). The expression level of *HSP70* were positively correlated with increasing exposure to high temperatures up to 48 h (Figure 3C) and declined to baseline after 24 h of recovery from heat shock stress. So far, heat shock proteins are known to be constitutively expressed in gill and liver tissues from Antarctic notothenioid fish [8,10], and we could identify that the *HSP70* was also constitutively expression, but not induced in liver or other tissues (brain, skin, kidney, and spleen) (Figures 2A and 3B). This was the first observation that the expression of HSR-related genes was induced under heat stress in the whole blood sample. Because cold stress, as well as heat stress, also can denature protein structures [37,39,46], we investigate whether the expression of several *HSP* genes is induced under cold shock stress at -2°C. We found that 46 genes were significantly upregulated more than two-fold under cold stress and 13 genes were also upregulated under heat shock stress, including *HSP* genes (Figure 3A,B,C, and Additional file 1: Table S15). Based on a GO enrichment test among genes upregulated more than two-fold under heat or cold stresses, the majority of enriched GO terms were shared between the two stressors (Additional file 1: Tables S16, S17 and Additional file 2: Figure S6). Shared GO terms resulting from shared genes upregulated under opposite stresses, such as heat and cold, were relevant in terms of protein stability. The induced HSR in Antarctic fish under thermal stress indicates that proteins were stable under normal conditions in blood. Based on tissue-specific gene expression analyses, genes related to the unfolded protein response (UPR) [47-49] with *HSP70*, *FKBP*, the 78-kDa glucose-regulated protein precursor (*GRP78*), *IRE1*, and transcription factor X-box binding protein 1 (*XBP1*), were also downregulated in whole blood compared to other tissues (brain, skin, liver, kidney, intestine, and spleen) (Additional file 2: Figure S7). The relative expression levels of genes related to the UPR support the stable states of blood proteins.

## Discussion

### The evolution of gene families in *N. coriiceps*

We investigated the size differences in gene family and calculated the dN/dS value to uncover evidence for evolution in Antarctic fish. We found significant contractions in 32 gene families, but could not identify significant expansions based on gene family evolution analysis. Although several genes (three solute carrier families and two glutamate receptors) of similar functions were found in contracted gene families (Additional file 3), we could not find significant GO terms in GO enrichment tests for the group of contracted gene families. We confirmed that *N. coriiceps* has the largest contractions of gene families among six fish. In dN/dS analyses comparing orthologs, our observation that the average dN/dS ratio of *N. coriiceps* genes was significantly higher than that of five other fish are supporting the conclusion of strong selection pressure affected the average dN/dS ratio of *N. coriiceps* (0.133). The constant low temperature around 1°C and higher oxygen solubility in the Southern Ocean would likely be strong selective factors on Antarctic fish in the 34 million years since Antarctica started to cool.

In GO enrichment tests with the most rapidly evolving 10% of *N. coriiceps* genes, we confirmed that most enriched GO terms were related with mitochondria. The average dN/dS ratio of 20 genes encoding mitochondrial proteins in *N. coriiceps* was significantly higher than that found in other five fish. Ten genes were directly related to oxidative phosphorylation; six genes encoded subunits of ATP synthase, one gene encoded a subunit of mitochondria complex III, and three genes encoded subunits of mitochondria complex IV. This observation indicates that rapid evolution in mitochondrial proteins might be related to adaptation under cold environment. Investigations on mitochondrial function of Antarctic fish showed that oxygen consumption rates and the coupling efficiency between electron transport and ATP synthesis were more sensitive to temperature than temperate fish [33-35]. The Arrhenius break temperature (ABT) reflects the adaptation temperature of the species, and it is the temperature at which there is a discontinuity in the slope of an Arrhenius plots of O_2_ consumption versus temperature. ABT for mitochondria of Antarctic fish is around 12°C much less than 20°C from several taxa of marine invertebrates and fish [33-35]. The temperature of acceptor control ratio (ACR), which is the ratio max rate adenosine diphosphate (ADP) induced O_2_ consumption to the basal rate in the absence of ADP, began to decrease also reflects the adaptation temperature. ACR decreased at approximate 18°C in the mitochondria of Antarctic fish in contrast to about 35°C in temperate fish [35]. Low ABT and the decrease of ACR at low temperature in the mitochondria of Antarctic fish is likely an adaptation to the cold and thermostable Southern Oceans, and might affect the rapid death of stenotherm, Antarctic fish, at a temperature around 10°C [34]. Oxygen transporter activity, including alpha and beta globins, among enriched GO terms were also related to the function of oxidative phosphorylation by efficiently supplying oxygen to mitochondria. Their rapid evolution might influence the phenomenon of thermal sensitivity of mitochondrial function in Antarctic fish and might be helpful to interpret the thermal limit of metabolic acclimation [50,51].

### Loss of PDSM in HSF1 and heat shock response in blood sample of *N. coriiceps*

In most organisms, heat shock responses are mediated mainly by heat shock factors. In the presence of stress, HSF1 complex dissociates to HSP90, HSP40, and HSP70, after that HSF1 have trimerized [52]. Trimeric HSF1 localizes to the nucleus and activates the transcription of target genes. During this process, HSF1 undergoes extensive PTMs in regulatory domain [41]. In humans, HSF1 contains a regulatory domain with three phosphorylation sites and one phosphorylation-dependent sumoylation motif (PDSM) [42], and sumoylation of HSF1 is inversely related to HSF1 activity [43,44]. HSF1 is maximally activated in the absence of sumoylation [43] (Figure 2C). During the attenuation phase, the transactivation of HSF1 is negatively regulated by elevated levels of both HSP40 (DNAJB1) and HSP70 [52]. The DNA-binding activity of HSF1 is regulated by acetylation of HSF1, and the expression of NAD^+^-dependent sirtuin (*SIRT1*) is negatively associated with acetylation of HSF1 (Figure 2B and C) [52]. We found that the major proteins regulating the HSR were expressed in blood and other tissues based on both RNA-seq and rt-PCR (Figure 2A) and the DNA-binding activity of HSF1 is reported in hepatocytes from *Trematomus bernacchii* (a common Antarctic notothenioid species) [36]. Sequence alignments also showed that *HSF1* of *N. coriiceps* contains conserved DNA binding domains, regulatory domains, and site of sequences related to trimerization. However, the *HSF1* of *N. coriiceps* does not contain an intact PDSM in the regulatory domain, but Asn is substituted for Ser in PDSM (KxExxSP) (Additional file 2: Figure S5). Consequently, HSF1 of *N. coriiceps* does not sumolyated by PTMs, which means the transactivation of HSP1 was not repressed by sumoylation. The absence of sumoylation in HSF1 maximizes more easily activated of HSF1 in the presence of stress (Figure 2C) [43]. We hypothesize that the HSR in Antarctic notothenioids is not constitutively activated to repair protein denaturation in constant cold environment, but readily responsive to the environment. Based on our transcriptomic experiments examining heat shock protein expression in various tissues subjected to thermal stress (Figure 3A), induction of the HSR was consistent with our hypothesis. The HSR in *N. coriiceps* has retained the ability to increase the expression of heat shock proteins (*HSP70*, *HSP ssb1*, and *HSP40*) at the transcriptional level in whole blood of *N. coriiceps* in response to acute thermal stress. Molecular evolution of alpha and beta globin, which comprise 21.2% of total transcripts in whole blood samples (Additional file 1: Table S18), might be related to this hypothesis. Because hemoglobin makes up about 40% of red blood cells in several red-blooded notothenioids [53], their rapid evolution might affect protein stability. Together with the presence of antifreeze glycoprotein in blood, which originated primarily from the exocrine pancreas and the stomach [3-5], our observation that HSR occurred in blood of *N. coriiceps* might be one of the evolution strategies to supply adequate oxygen in cold environment.

### Tissue-specific gene expression patterns related to HSR and UPR

Tissue-specific gene expression patterns related to the HSR showed that the expression of *FKBP* and *HSP70* were decreased in whole blood samples compared to other tissues in normal condition (Additional file 2: Figure S10). The heat shock response is known to occur in the cytosol, so we also examined genes associated with UTR in the endoplasmic reticulum (ER) (Additional file 2: Figure S7) [47,49]. *GRP78*, *IRE1*, and *XBP1* were downregulated in blood. The UPR is typically triggered in response to accumulation of misfolded proteins in the lumen of the ER, after GRP78 is released from IRE1 to support proper protein folding [49]. IRE1 through autophosphorylation activates its ribonuclease domain and catalyzes the excision of unconventional introns from ubiquitously expressed XBP1 [48]. This excision causes a frame shift in the *XBP1* coding sequence, resulting in the production of the 376-amino acid XBP1 protein. Active XBP1 upregulates genes involved in UPR [47,49]. These results support our conclusion that blood proteins in cytosol and in ER have the stability which do not induced the constitutive HSR.

## Conclusions

In this study, we provide the first annotated genome of an Antarctic species that dominates the fish fauna of the Southern Ocean and shows remarkable adaptations to cold temperatures. The genome sequence of *N. coriiceps* increases our understanding of the evolution trajectory of some major life-history traits of these Antarctic fish. We demonstrated that *N. coriiceps* have rapidly evolved mitochondrial proteins and hemoglobin, and have preserved the HSR in blood. Our observations were associated with oxidative phosphorylation in aerobic cellular respiration and might make a contribution to adapt to an extremely cold environment through the proper function of aerobic cellular respiration. Our study provides a reference genome for use in future comparative studies of Antarctic adaptations and can be applied to ecological and population studies of Antarctic biota.

## Materials and methods

### Ethics statement

This study including sample collection and experimental research conducted on these animals was according to the law on activities and environmental protection to Antarctic approved by the Minister of Foreign Affairs and Trade of the Republic of Korea.

### DNA library construction and sequencing

*N. coriiceps* (length 35 cm) were collected from depths of 20 to 30 m in Marian Cove, near King Sejong Station, on the northern Antarctic Peninsula (62°14′S, 58°47′W) in January 2012 using the hook-and-line method, and water temperatures were monitored at 1.6 ± 0.8°C in January 2012. High-molecular-weight genomic DNA from *N. coriiceps* was extracted using the Gentra Puregene Blood Kit (Qiagen). For Illumina Hiseq 2000 sequencing, five library types were constructed with 150, 300, 350, 500, and 600 bp sheared genomic DNA, and subsequently prepared using the standard Illumina sample preparation methods. Mate-pair libraries (3, 7, and 20 kb) for the GS-FLX titanium apparatus were prepared for scaffolding, and sequencing was performed according to the manufacturer’s instructions (Additional file 1: Table S1). All sequencing processes were performed by DNA Link, Inc. (Additional file 1: Table S2).

### Genome assembly using Celera assembly

Hybrid assemblies were performed using the Celera Assembler (Ver. 7.0) with Illumina short reads and 454 reads [21]. Prior to assembly, Illumina reads were trimmed using the FASTX-Toolkit (Ver. 0.0.11) [54] with the parameters -t 20, -l 70, and -Q 33, after which a paired sequence from trimmed Illumina reads was selected. Finally, read data with 110-fold coverage were obtained. Among the final read data, 74× trimmed Illumina reads with various insert sizes (150, 350, 500, and 600 bp) were randomly selected due to memory limitation on the available linux machine, and converted to the FRG file format (required by the Celera assembler) using FastqToCA. Using sffToCA, 1.8 × 454 reads were converted to the FRG file format by removing a linker sequence from 454 reads generated using GS-FLX. Assembly was performed on a 96-processor workstation with Intel Xeon X7460 2.66 GHz processors and 1 terabyte RAM with the parameters overlapper = ovl, unitigger = bogart, utgGraphErrorRate = 0.03, utgGraphErrorLimit = 2.5, utgMergeErrorRate = 0.030, utgMergeErrorLimit = 3.25, dovlErrorRate = 0.1, cnsErrorRate = 0.1, cgwErrorRate = 0.1, merSize = 22, and doOverlapBasedTrimming = 1. The initial Celera assembly had a total size of 602 Mb, N50 Contig size of 8,581 bp, and N50 scaffold size of 219 kb with 88,548 gaps (18 Mb). The size distributions of the Celera contigs were plotted and the assembled contig revealed a contig coverage of approximately 33× (Additional file 1: Table S3 and Additional file 2: Figure S1).

### Error correction of PacbioRS reads

The genome was sequenced using PacbioRS, which can generate continuous long reads (CLRs) of up to 10 kb in length, and can be used to upgrade draft genomes containing gaps using PBJelly (Ver. 12.9.14) [22]. However, CLRs show only 82.1% to 84.4% base accuracy [55]. Thus, error correction was performed using the command pacBioToCA [56] with the parameters -length 500, -partitions 200, -shortReads, -l NC, -t 20, and -s pacbio.spec. Illumina (50× read coverage of genome) reads were used for correction. Illumina reads were trimmed using FASTX-Toolkit [56] with the parameters -t 20, -l 50, and -Q 33. Pacbio.spec files specified the parameters for overlapping Illumina and pacbio data for correction: utgErrorRate = 0.25, utgErrorLimit tgErrorLcnsErrorRate = 0.25, cgwErrorRate = 0.25, ovlErrorRate = 0.25, and merSize = 10. After correction, pacBio-corrected reads were analyzed using FastQC [57]. A total of 2,640,379 CLRs (7.6× read coverage of genome) were used for error-correction, which generated 2,415,333 error-corrected reads (2.3× read coverage of genome) (Additional file 1: Table S1). The average CLR length decreased from 1,819 to 969 bp. The resulting error-corrected CLRs were used for gap filling.

### Gap filling

Gap filling was conducted in two stages. Initially, we closed gaps using the Gapfiller Ver.1.9 software with 116× trimmed Illumina reads with default settings [23,58]. The remaining gaps of the scaffold from Gapfiller were closed using error-corrected CLRs from PacbioRS using the PBJelly software (Ver. 12.9.14) with the parameter of minGap = 10 [22]. Using Gapfiller, 18,400 gaps (2.3 Mb in length) were closed and 7,394 gaps (3.0 Mb) were filled with error-corrected CLRs. A total of 25,794 gaps were closed (closed gap size of 5.3 Mbases). After gap filling, the number of scaffolds decreased from 11,622 to 8,155 and the N50 contig size increased from 8,518 bases to 11,563 bases (Additional file 1: Table S3).

### Repeat analysis

We constructed a *de novo* repeat library using RepeatModeler (Ver. 1.0.3) [59], including the RECON (Ver. 1.07) [59] and RepeatScout (Ver. 1.0.5) [60] software, with default parameters. Consensus sequences and classification information for each repeat family were generated, and tandem repeats including simple repeats, satellites, and low complexity repeats were predicted using TRF [61].

### Assembly validation

The *N. coriiceps* BAC library was obtained from the Children’s Hospital Oakland Research Institute (BAC library ID, VMRC-19). We sequenced six BAC clones using GS-FLX and assemblies were performed using the Celera Assembler (Ver. 7.0). Six sequenced BAC clones were aligned to the assembled genome scaffolds using NUCmer (Ver. 3.07) with default settings. Mummerplot (Ver. 3.5) was used with the NUCmer delta file as input [62] (Additional file 2: Figure S2).

### Transcriptome assembly

Total RNA from seven tissues (brain, skin, egg, kidney, muscle, stomach, and blood) was prepared using the Qiagen kit according to the manufacturer’s instructions. The quality of total RNA was confirmed on an Agilent Bioanalyzer™. Library construction and sequencing were performed using DNAlink with an Illumina HiSeq 2000 System and PacbioRS. A total of 36,046 Mbases and 300 Mbases were obtained using the two methods, respectively (Additional file 1: Table S4). The transcriptome sequence reads were mapped to the *N. coriiceps* genome using the publicly available packages Bowtie (Ver. 0.12.9) [63,64], TopHat (Ver. 2.0.6) [65,66], and Cufflinks (Ver. 2.0.2) [67-69] (Additional file 1: Table S5). PacbioRS reads from each tissue (egg, skin, and muscle) were error-corrected with Illumina paired-end reads of mRNAs corresponding to each tissue [56] (see the Error correction of PacbioRS reads and Additional file 1: Table S5). Transcript assemblies with Cufflinks and error-corrected CLR were both used for gene annotation.

### Gene annotation (MAKER)

We used MAKER2 for genome annotation [70]. MAKER is a portable and easily configurable genome annotation pipeline. Maker first identified repetitive elements using RepeatMasker (Ver. 3.3.0) [71]. This masked genome sequence was used for *ab initio* gene prediction with the SNAP software [72], after which alignment of expressed sequence tags with BLASTn and protein information from tBLASTx were included. We used the *de novo* repeat library of *N. coriiceps* from RepeatModeler (Ver. 1.0.5) for RepeatMasker (Ver. 3.3.0); proteins from five fish species with data from Ensembl release 69 (*D. rerio*, *G. aculeatus*, *T. rubripes*, *T. nigroviridis*, and *G. morhua*) were included in the analysis. Transcriptome assembly results were used for expressed sequence tags. Next, MAKER polished the alignments using the program Exonerate, which provided integrated information to synthesize SNAP annotation. MAKER then selected and revised the final gene model considering all information. A total of 32,661 transcripts and 32,260 genes were predicted using MAKER in *N. coriiceps*, and 93,090 *ab initio* gene predictions were generated. Additionally, 29,045 out of 32,260 genes were assigned preliminary functions based on automated annotation using Blast2Go (Ver. 2.6.0) [73].

### Non-coding RNA

The Infernal software package (Ver. 1.1) [74] and CMs from the Rfam database [75] were used to identify non-coding RNAs in the *N. coriiceps* scaffolds (Additional file 1: Table S8). We identified putative tRNA genes using tRNAscan-SE (Ver. 1.21) [76]. tRNAscan-SE uses a covariance model (CM) that scores candidates based on their sequence and predicted secondary structures (Additional file 1: Table S9).

### Ortholog analysis

We identified orthologous groups using OrthoMCL (Ver. 2.0.5) [77], which generated a graphical representation of sequence relationships that was then divided into subgraphs using the Markov Clustering Algorithm (MCL) from multiple eukaryotic genomes [77]. We used the standard parameters and options of OrthoMCL for all steps. In this analysis, six fish genomes (*D. rerio*, *G. aculeatus*, *T. rubripes*, *T. nigroviridis*, *G. morhua*, and *N. coriiceps*) were used, with coding sequences collected from Ensemble release 69 except for *N. coriiceps* (Additional file 2: Figure S4). For *N. coriiceps*, the coding sequence from the MAKER annotation pipeline was used.

### Likelihood analysis of gene gain and loss

To estimate the average gene gain/loss rate and to identify gene families that have undergone significant size changes, we used the program CAFE3.0 [26,27,29,78]. The phylogenetic tree of the species drawn with Timetree [79] was used for analysis. We performed the program using *P* <0.05, estimated birth (*λ*) and death (μ) rates by using the program lambdamu with ‘-s’ option. We calculated the number of gene gains and losses on each branch of the tree with the ‘-t’ option. Using *P* <0.0001, we expect there to be approximately one significant result by chance and calculated the exact *P* values for transitions over every branch. We called individual branches significant at *P* <0.005 [29].

### dN/dS analysis

We first identified orthologous groups using OrthoMCL for dN/dS analysis. Six fish genomes (*D. rerio*, *G. aculeatus*, *T. rubripes*, *T. nigroviridis*, *G. morhua*, and *N. coriiceps*) were used for analysis, and coding sequences from five genomes were collected from Ensembl release 69. We identified 8,974 orthologous groups common to all six fish (Additional file 1: Table S10). To establish sets of othologs among six fish, the method of reciprocal best hits using BLASTp was used. Protein-coding sequences of orthologs were aligned using PRANK (Ver. 130820) under a codon model [80], and poor alignment sites were eliminated using Gblock (Ver. 0.91) under a codon model [81]. Poor alignment sequences were also eliminated (below 50% similarity in length and 40% in identity). Codeml in the Phylogenetic Analysis by Maximum Likelihood (PAML) package (Ver. 4.7a) was used to estimate the dN (the rate of non-synonymous substitutions), dS (the rate of synonymous substitutions) and the ratio of dN/dS using the branch model (model = 2, NSsites = 0, fix_omega = 0) and basic model (model = 0, NSsites = 0, fix_omega = 0) under F3X4 codon frequency and codon sequence types [82]. The species tree was calculated by using PHYLIP’s dnaml (Ver. 3.695). To identify whether the dN/dS in each lineage is different from the rest of tree, a Likelihood Ratio Test (LRT) of branch model to basic model was performed, and false discovery rate (FDR) was used to control the *P* values in multiple tests. Additionally, we performed a LRT of a branch model to a model of neutrality (model = 2, NSsite = 0, fix_omega = 1) and FDR was also used to adjust the *P* value [82]. Orthologs with dS >3 or tanssition/tranversion ratio >10 were filtered. Finally, dN/dS of 5,039 single-copy gene orthologs for the six fish was determined.

### Functional analysis of rapidly evolving genes

dN was considered as the indicator to distinguish whether a protein rapidly evolved or not, because highly expressed genes may result in underestimates of the synonymous substitution rate even with likelihood methods [32]. To investigate whether any functional categories were statistically over-represented among rapidly evolving *N. coriiceps* genes (comprising the fastest evolving 10% of total genes, 505 genes in all) in terms of dN [30,32], we applied AgriGO [31], a web-based tool for gene ontology analysis, with significant levels of *P* = 0.05. Complete hierarchies of GO terms for each gene were examined.

### Gene expression under temperature stress

*N. coriiceps* were transported in insulated containers with aerated sea water to the King Sejong Station, and were acclimated in large tanks circulating with fresh sea water at +2.0 ± 0.2°C at least 3 days prior to experiments. We prepared two other large tanks at -2°C, 2°C, and 4°C for cold stress, control, and heat stress, respectively. After acclimation, three groups of nine specimens each of *N. coriiceps* were kept in a cold tank, a normal tank, and a heated tank with aerated sea water. Three groups of three specimens of *N. coriiceps* each were sacrificed at 0, 24, and 48 h after stress. We then dissected each tissue (brain, skin, egg, kidney, muscle, and stomach) of *N. coriiceps*. Before dissection, blood samples were collected from the brachial vein using a sterile 3 mL syringe. Dissected tissues were lysed, immersed in RNAlater, and stored at -70°C for future experiments.

For RNA-Seq experiments, we prepared mRNA from blood samples from three specimens of each individual sample at each temperature condition. Sequencing was performed with Illumina Hiseq 2000, and generated reads were trimmed using sickle (Ver. 1.2) with approximately 75 bases in length and approximately 20 in base quality (Additional file 1: Table S13). Trimmed reads of each tissue were mapped to the annotated scaffold of the *N. coriiceps* genome using TopHat (Ver. 2.0.6) [66], and differentially expressed genes were assessed using Cuffdiff (Ver. 2.0.2) [69]. Cuffdiff compares FPKM (fragments per kilobase of exon per million fragments mapped) values between each sample and calculates fold changes in expression for each gene based on statistical significance (cutoff, *P* ≤0.05) (Additional file 1: Table S14 and Additional file 5).

### Tissue-specific gene expression

Illumina paired-end reads of each tissue were mapped to the annotated scaffold of *N. coriiceps* genome using TopHat (Ver. 2.0.6) [66], and differentially expressed genes were assessed using Cuffdiff (Ver. 2.0.2) [69] (cutoff, *P* ≤0.05).

### Statistical analysis

Comparisons of multiple samples were made by an analysis of variances (ANOVA) with Bonferroni post hoc test. The Statistical Package for the Social Sciences software (SPSS) was used for analyses.

#### Accession codes

The *N. coriicpes* has been deposited at BioProject: 66471, and the whole-genome shotgun project has been deposited at DDBJ/EMBL/GenBank under accession AZAD00000000. This paper describes the first version, AZAD01000000. Raw RNA sequencing reads have been submitted to the NCBI Sequence Read Archive database (SRA091269).

## Additional files

Additional file 1: Table S1. Statistics for each DNA library. Ten categories of DNA libraries with various insert sizes for three platform sequencers were constructed. **Table S2:** Insert size of each paired-end libraries. The range of paired-end insert sizes was estimated by mapping the reads onto the assembled genome sequence. **Table S3:** Statistics of genome assembly and gap filling. **Table S4:** Sequencing statistics of transcriptome analysis of each organ of *N. coriiceps* using two sequencer platforms. **Table S5:** Assembly results of transcriptome analysis of each organ of *N. coriiceps.* **Table S6:** Genome annotation statics. **Table S7:** General statistics of gene in *N. coriiceps*. **Table S8:** Known repetitive and transposable elements in the *N. coriiceps* genome. **Table S9:** Number of tRNA in the *N. coriiceps* nuclear genome. **Table S10:** Shared orthologous gene clusters among six fishes. For genes with multiple alternative transcripts, the transcript with the best alignment was selected. Genes with lengths less than 100 bp were discarded. **Table S11:** GO terms over-represented in dN/dS analysis. **Table S12:** Gene which is included in GO terms over-represented in dN/dS analysis. **Table S13:** Sequencing reads used in analysis of RNA-Seq under stresses. **Table S14:** The result of RNA-seq. Detailed gene lists are shown in Tables S16 - S22. **Table S15:** Upregulated genes in blood under both cold and heat stress. **Table S16:** GO enrichment test in blood under heat stress. **Table S17:** GO enrichment test in blood under cold stress. **Table S18:** Top blood-specific genes and their transcript percentages in whole blood transcriptomes. **Table S19:** Downregulated genes in blood under cold stress. **Table S20:** Downregulated genes in blood under heat stress. **Table S21:** Downregulated genes in blood under both cold and heat stress. **Table S22:** Shared genes in downregulated group under heat and cold stress in the brain. Additional file 2: Figure S1. Contig characteristics. **(A)** The contig length distribution shows that small size contig were incorporated into other contig. **(B)** Contig coverage. **Figure S2:** Comparison of the assembled genome with six BACs sequences. NUCmer alignments of the Celera genome assembly scaffolds (x-axis) and BAC sequences (y-axis) ordered and oriented such that the largest hits cluster. **Figure S3:** Gene ontology (GO) distribution after BlastX analysis for *N. coriiceps* transcriptome sequences as grouped by biological process (red), cellular component (cyan), and molecular function (grey). **Figure S4:** Cladogram representing phylogenetic relationship between selected 5,039 orthologous genes of six fishes. **Figure S5:** Sequence alignment of heat shock factor-1. The sites required for post-translational modification were well-conserved except Ser303, corresponding to human *HSF1* in PDSM in Antarctic fish (*N. coriiceps*, Dragon fish and Icefish). **Figure S6:** Significantly over-represented GO terms under heat and cold stress among upregulated genes from blood. **Figure S7:** Tissue-specific expression of genes related to the HSR. **(A)** HSR and UPR were confirmed to be downregulated with RNA-Seq analysis in whole blood sample. **(B)** FKBP, HSP70, and HSP40 genes were confirmed to be downregulated in blood samples more than other tissues in HSR. XBP1 gene, the representative transcription factor in UPR, was also downregulated in blood sample. **Figure S8:** Significantly over-represented GO terms under heat and cold stress among downregulated genes from blood and gene expression included in heme binding (GO:0020037). **(A,B)** Significantly over-represented GO terms among the downregulated genes from blood showed the similar GO terms hierarchy. Five GO terms were shared in both cold and heat stress. **(C)** Results of RNA-Seq studies show the expression-pattern of genes included in GO:00200037. **Figure S9:** Toxicity of nitric oxide and superoxide. **Figure S10:** Forty-one GO terms focused on seven GO terms in the gene ontology hierarchy. Additional file 3:
**Significant contraction.** Thirty-two families showing significantly difference in *N. coriiceps* lineage. The program CAFE3.0 was used to identify gene families that have undergone significant size changes using *P* <0.0001. Additional file 4:
**Rapid evolving gene.** dN was considered as the indicator to distinguish whether a protein rapidly evolved or not, and 505 genes comprising the fastest evolving 10% of total genes were selected with dN. A total of 505 genes were denoted as R in rapid evolving gene (column D), *N. coriiceps* lineage-specific genes were denoted as D, and genes under positive selection were denoted as PS. Additional file 5:
**Gene expression under temperature stress.**
