# Supplementary material for: The genome sequence of the Antarctic bullhead notothen reveals evolutionary adaptations to a cold environment
Source: Genome Biol. 2014 Sep 25;15(9):468. doi: 10.1186/s13059-014-0468-1 (PMC4192396; doi:10.1186/s13059-014-0468-1)
Supplement: Additional file 1: Table S1. — Statistics for each DNA library. Ten categories of DNA libraries with various insert sizes for three platform sequencers were constructed. Table S2: Insert size of each paired-end libraries. The range of paired-end insert sizes was estimated by mapping the reads onto the assembled genome sequence. Table S3: Statistics of genome assembly and gap filling. Table S4: Sequencing statistics of transcriptome analysis of each organ of N. coriiceps using two sequencer platforms. Table S5: Assembly results of transcriptome analysis of each organ of N. coriiceps. Table S6: Genome annotation statics. Table S7: General statistics of gene in N. coriiceps. Table S8: Known repetitive and transposable elements in the N. coriiceps genome. Table S9: Number of tRNA in the N. coriiceps nuclear genome. Table S10: Shared orthologous gene clusters among six fishes. For genes with multiple alternative transcripts, the transcript with the best alignment was selected. Genes with lengths less than 100 bp were discarded. Table S11: GO terms over-represented in dN/dS analysis. Table S12: Gene which is included in GO terms over-represented in dN/dS analysis. Table S13: Sequencing reads used in analysis of RNA-Seq under stresses. Table S14: The result of RNA-seq. Detailed gene lists are shown in Tables S16 - S22. Table S15: Upregulated genes in blood under both cold and heat stress. Table S16: GO enrichment test in blood under heat stress. Table S17: GO enrichment test in blood under cold stress. Table S18: Top blood-specific genes and their transcript percentages in whole blood transcriptomes. Table S19: Downregulated genes in blood under cold stress. Table S20: Downregulated genes in blood under heat stress. Table S21: Downregulated genes in blood under both cold and heat stress. Table S22: Shared genes in downregulated group under heat and cold stress in the brain. [file 13059_2014_468_MOESM1_ESM.docx]

**Additional file 1. Table S1 – Table S22.**

**Table S1. Statistics for each DNA library.** Ten categories of DNA libraries with various insert sizes for three platform sequencers were constructed.

| **Library** |  | **Num. of Reads** | **Num. of Sequences (bp)** | **Num. of Reads  (Trimmed)** | **Num. of Sequences (Trimmed)** | **Num. of Reads (Trimmed&Used)** | **Num. of Sequences (Trimmed & Used)** |
| --- | --- | --- | --- | --- | --- | --- | --- |
| **Continuous Long Read** | **Sum** | **2,640,379** | **4,805,268,872** | **2,415,333** | **2,318,055,976** | **2,415,333** | **2,318,055,976** |
|  | Single | 2,640,379 | 4,805,268,872 | 2,415,333 | 2,318,055,976 | 2,415,333 | 2,318,055,976 |
| **GS-FLX Paired-End** | **Sum** | **5,065,150** | **1,666,509,204** | **7,744,289** | **2,004,270,538** | **5,730,680** | **1,185,467,442** |
|  | 20Kbp | 2,498,260 | 786,807,325 | 3,651,379 | 961,518,075 | 2,562,596 | 519,300,012 |
|  | 3Kbp | 1,394,475 | 497,726,608 | 2,219,982 | 588,231,416 | 1,717,362 | 373,634,593 |
|  | 8Kbp | 1,172,415 | 381,975,271 | 1,872,928 | 454,521,047 | 1,450,722 | 292,532,837 |
|  | Single | 1,817,904 | 590,522,747 | 2,276,549 | 819,568,063 | 2,276,549 | 819,568,063 |
| **Illumina Paired-End** | **Sum** | **854,317,848** | **216,986,699,496** | **750,458,264** | **69,977,287,701** | **498,912,642** | **47,163,704,155** |
|  | 150bp | 61,274,520 | 12,377,453,040 | 100,000,000 | 9,878,047,259 | 100,000,000 | 9,878,047,259 |
|  | 300bp | 109,793,542 | 22,178,295,484 | 107,380,330 | 10,195,484,757 | 81,144,366 | 7,807,711,630 |
|  | 350bp | 76,775,928 | 23,186,330,256 | 62,310,018 | 5,852,739,210 | 62,310,018 | 5,852,739,210 |
|  | 500bp | 239,104,844 | 48,299,178,488 | 219,171,362 | 20,557,364,296 | 90,915,794 | 8,661,654,674 |
|  | 600bp | 367,369,014 | 110,945,442,228 | 261,596,554 | 23,493,652,179 | 164,542,464 | 14,963,551,382 |
| **Total** |  | **863,841,281** | **224,049,000,319** | **762,894,435** | **75,119,182,278** | **509,335,204** | **51,486,795,636** |
| **Coverage (folds)**^1^ |  |  | **373.4** |  | **125.2** |  | **85.8** |

^1^Coverage is defined as the number of sequences divided by the genome size.

**Table S2. Insert size of each paired-end libraries.** The range of paired-end insert sizes was estimated by mapping the reads onto the assembled genome sequence.

| **Library** |  | **Average of insert size (bases)**^1^ | **Standard deviation of insert size (+/-)**^2^ |
| --- | --- | --- | --- |
| **GS-FLX Paired-End** | 20Kbp | 18,527.6 | 4,631.9 |
|  | 3Kbp | 2,665.7 | 769.8 |
|  | 8Kbp | 7,752.3 | 1,938.1 |
| **Illumina Paired-End** | 150bp | 167.9 | 14.0 |
|  | 300bp | 335.4 | 13.9 |
|  | 350bp | 390.5 | 13.3 |
|  | 500bp | 532.9 | 22.1 |
|  | 600bp | 624.8 | 20.6 |

^1^Average of insert size reported by the Celera assembly program after assembly.

^2^Starndard deviation of insert size reported by the Celera assembly program after assembly.

**Table S3. Statistics of genome assembly and gap filling.**

|  |  | **Celera assembler (Version : 7.0)** | **GAPfiller (Version : 1.9)** | **Pbjelly (Version : 12.9.14)** |
| --- | --- | --- | --- | --- |
| **Scaffold** | total scaffold length (bases) | 602,295,735 | 636,049,014 | 636,793,288 |
|  | the number of gaps | 88,548 | 76,150 | 62,754 |
|  | gap size (bases) | 18,468,690 | 14,441,127 | 13,131,591 |
|  | the number of scaffolds | 38,062 | 38,062 | 38,062 |
|  | N50 contig length (bases) | 188,605 | 216,434 | 219,067 |
|  | Max scaffold length (bases) | 28,707,441 | 28,717,475 | 28,796,675 |
|  |  |  |  |  |
| **Contig** | total contig length (bases) | 583,827,045 | 621,614,170 | 623,701,534 |
|  | the number of contigs | 127,598 | 115,200 | 100,606 |
|  | N50 contig length (bases) | 8,581 | 10,575 | 11,563 |
|  | Max contig length (bases) | 120,777 | 184,241 | 226,807 |

**Table S4. Sequencing statistics of transcriptome analysis of each organ of *N. coriiceps* using two sequencer platforms.**

|  |  | **Blood** | **Egg** | **Skin** | **Kidney** | **Muscle** | **Stomach** | **Brain** |
| --- | --- | --- | --- | --- | --- | --- | --- | --- |
| Illumina HiSeq 2000 | Total num. of reads | 8,335,794 | 93,923,026 | 67,504,452 | 60,161,028 | 88,281,852 | 5,077,628 | 33,616,256 |
|  | Total num. of bases | 841,915,194 | 9,486,225,626 | 6,817,949,652 | 6,076,263,828 | 8,916,467,052 | 512,840,428 | 3,395,241,856 |
|  | Num. trimmed reads | 8,335,794 | 50,000,000 | 67,501,898 | 60,161,028 | 60,000,000 | 5,077,628 | 33404860 |
|  | Num. of trimmed bases | 841,915,194 | 5,050,000,000 | 6,817,691,698 | 6,076,263,828 | 6,060,000,000 | 512,840,428 | 3,373,890,860 |
| Pacbio RS | Total num. of reads |  | 160,667 | 128,900 |  | 118,476 |  |  |
|  | Total num. of bases |  | 142,223,519 | 114,991,009 |  | 103,676,744 |  |  |
|  | Num. error-corrected reads |  | 236,653 | 188,395 |  | 178,267 |  |  |
|  | Num. error-corrected bases |  | 119,291,958 | 96,875,119 |  | 84,127,846 |  |  |

**Table S5. Assembly results of transcriptome analysis of each organ of *N. coriiceps.***

| **Platform** | **Program** | **Tissue** | **Total num. of contigs** | **Total num. of bases** | **Average size of contigs** |
| --- | --- | --- | --- | --- | --- |
| GS-FLX | Newbler  (Version : 2.5.3) | Brain | 9,532 | 5,547,624 | 582 |
|  |  | Liver | 24,510 | 23,676,660 | 966 |
| Illumina HiSeq | TOPHAT  (Version : 2.0.6) | Blood | 14,848 | 17,759,115 | 1,196 |
|  |  | Egg | 29,954 | 41,949,443 | 1,400 |
|  |  | Kidney | 37,154 | 53,429,245 | 1,438 |
|  |  | Muscle | 23,897 | 31,883,937 | 1,334 |
|  |  | Stomach | 5,663 | 4,147,701 | 732 |
|  |  | Brain | 39,133 | 67,645,262 | 1,728 |
|  |  | Skin | 39,168 | 55,031,213 | 1,405 |
| Pacbio RS | PacbioToCA | Egg | 236,653 | 119,291,958 | 504 |
|  |  | Skin | 188,395 | 96,875,119 | 514 |
|  |  | Muscle | 178,267 | 84,127,846 | 472 |

**Table S6. Genome annotation statics.**

|  | **Number** | **Base (base)** | **Assembly (%)** |
| --- | --- | --- | --- |
| Protein coding genes | 32,260 | 47,712,235 | 7.4926% |
| rRNA | 59 | 6,460 | 0.0010% |
| miRNA | 1,740 | 171,141 | 0.0269% |
| snoRNA | 282 | 33,539 | 0.0053% |
| snRNA | 57 | 6,460 | 0.0010% |
| tRNA | 512 | 40,349 | 0.0063% |
| miscRNA | 28 | 3,186 | 0.0005% |
| Sum | 34,938 | 47,973,370 | 7.5336% |

**Table S7. General statistics of gene in *N. coriiceps*.**

|  | **Number** |
| --- | --- |
| The number of genes | 32,260 |
| The average mRNA length (bp) | 1,478 |
| The number of exons | 214,650 |
| The number of exons per genes | 6.65 |
| The average coding sequence length (bp) | 1,063 |

**Table S8. Known repetitive and transposable elements in the *N. coriiceps* genome.**

|  |  | ***de novo* repeat** | |
| --- | --- | --- | --- |
|  | **TE element** | **Length** | **%** |
| Retrotransposon | SINE^1^ | 1,873,487 | 0.29% |
|  | LINE^2^ | 13,262,368 | 2.08% |
|  | LTR^3^ | 2,282,209 | 0.36% |
| DNA transposon | | 35,353,051 | 5.55% |
| Unknown | | 46,670,060 | 7.33% |
| Tandem repeat | Satellites | 528,057 | 0.08% |
|  | Simple repeats | 11,962,350 | 1.88% |
| Low complexity | | 3,629,184 | 0.57% |
| Total | | 115,560,766 | 18.15% |

^1^SINE : short interspersed nuclear element

^2^LINE : long interspersed nuclear element3

^3^LTR : long terminal repeat

**Table S9. Number of tRNA in the *N. coriiceps* nuclear genome.**

|  | **Number of tRNA** |
| --- | --- |
| Ala | 34 |
| Arg | 61 |
| Asn | 9 |
| Asp | 8 |
| Cys | 17 |
| Gln | 20 |
| Glu | 37 |
| Gly | 23 |
| His | 4 |
| Ile | 26 |
| Leu | 48 |
| Lys | 27 |
| Met | 20 |
| Phe | 17 |
| Pro | 25 |
| Pseudo | 3 |
| SeC(e) | 1 |
| Ser | 54 |
| Thr | 48 |
| Trp | 15 |
| Tyr | 21 |
| Undet | 1 |
| Val | 10 |
| Sum | 529 |

**Table S10. Shared orthologous gene clusters among six fishes.** For genes with multiple alternative transcripts, the transcript with the best alignment was selected. Genes with lengths less than 100 bp were discarded.

| **Group**^1^ | **Number** |  | **Group** | **Number** |
| --- | --- | --- | --- | --- |
| **A** | **3,820** |  | NFTZS | 366 |
| AS | 501 |  | NFZ | 89 |
| **F** | **6,234** |  | NFZA | 46 |
| FA | 47 |  | NFZAS | 502 |
| FAS | 77 |  | NFZS | 81 |
| FS | 181 |  | NS | 249 |
| FT | 328 |  | NT | 206 |
| FTA | 24 |  | NTA | 11 |
| FTAS | 180 |  | NTAS | 82 |
| FTS | 108 |  | NTS | 35 |
| FTZ | 137 |  | NTZ | 116 |
| FTZA | 65 |  | NTZA | 34 |
| FTZAS | 1,680 |  | NTZAS | 274 |
| FTZS | 200 |  | NTZS | 44 |
| FZ | 189 |  | NZ | 453 |
| FZA | 35 |  | NZA | 104 |
| FZAS | 190 |  | NZAS | 320 |
| FZS | 60 |  | NZS | 108 |
| **N** | **13,123** |  | **S** | **3,799** |
| NA | 133 |  | **T** | **2,642** |
| NAS | 251 |  | TA | 24 |
| NF | 176 |  | TAS | 38 |
| NFA | 12 |  | TS | 39 |
| NFAS | 86 |  | TZ | 91 |
| NFS | 48 |  | TZA | 28 |
| NFT | 78 |  | TZAS | 117 |
| NFTA | 43 |  | TZS | 34 |
| NFTAS | 426 |  | **Z** | **15,524** |
| NFTS | 131 |  | ZA | 144 |
| NFTZ | 178 |  | ZAS | 148 |
| NFTZA | 158 |  | ZS | 118 |
| **NFTZAS** | **8,974** |  |  |  |

^1^Z:*Danio rerio*, S:*Gasterosteus aculeatus*, F:*Takifugu rubripes*, T:*Tetraodon nigroviridis*, A:*Gadus morhua* and N: *Notothenia coriiceps*

**Table S11. GO terms overrepresented in dN/dS analysis.**

| GO term | Ontology | Description | *p*-value | FDR |
| --- | --- | --- | --- | --- |
| GO:0006119 | P | oxidative phosphorylation | 7.9E-10 | 2.8E-07 |
| GO:0015986 | P | ATP synthesis coupled proton transport | 4.1E-08 | 4.8E-06 |
| GO:0015992 | P | proton transport | 0.000015 | 0.001 |
| GO:0006818 | P | hydrogen transport | 0.000015 | 0.001 |
| GO:0006754 | P | ATP biosynthetic process | 0.00036 | 0.018 |
| GO:0015078 | F | hydrogen ion transmembrane transporter activity | 4.2E-08 | 5.4E-06 |
| GO:0016491 | F | oxidoreductase activity | 0.00047 | 0.015 |
| GO:0004129 | F | cytochrome-c oxidase activity | 0.00095 | 0.015 |
| GO:0004364 | F | glutathione transferase activity | 0.002 | 0.025 |
| GO:0050254 | F | rhodopsin kinase activity | 0.002 | 0.025 |
| GO:0005344 | F | oxygen transporter activity | 0.0041 | 0.048 |
| GO:0000276 | C | mitochondrial proton-transporting ATP synthase complex, coupling factor F(o) | 2.1E-07 | 0.000012 |
| GO:0045263 | C | proton-transporting ATP synthase complex, coupling factor F(o) | 1.4E-06 | 0.000032 |
| GO:0005743 | C | mitochondrial inner membrane | 0.000011 | 0.00016 |
| GO:0005739 | C | mitochondrion | 0.000057 | 0.00064 |
| GO:0033177 | C | proton-transporting two-sector ATPase complex, proton-transporting domain | 0.00011 | 0.00087 |
| GO:0042612 | C | MHC class I protein complex | 0.0002 | 0.0012 |

**Table S12. Gene which is included in GO terms overrepresented in dN/dS analysis.**

|  | **Locus tag** | **Gene** | **Specification** | **Rapid evolving gene^1^** | ***N. coriiceps* linegie-specific gene^2^** | **Gene under positive selection^3^** |
| --- | --- | --- | --- | --- | --- | --- |
| 1 | GEY_24462 | G protein-coupled receptor kinase 7B-like | | R | D | PS |
| 2 | GEY_12112 | beta globin (hbb2) | blood | R |  |  |
| 3 | GEY_14631 | alpha globin (hba2) | blood | R |  |  |
| 4 | GEY_18816 | beta-2 microglobulin | blood | R |  |  |
| 5 | GEY_13734 | ATP synthase-coupling factor 6, mitochondrial | mitochondrial | R | D |  |
| 6 | GEY_21250 | ATP synthase subunit g, mitochondrial | mitochondrial | R |  |  |
| 7 | GEY_09861 | ATP synthase subunit d, mitochondrial | mitochondrial | R |  |  |
| 8 | GEY_21396 | ATP synthase subunit b, mitochondrial | mitochondrial | R | D | PS |
| 9 | GEY_17898 | cytochrome c oxidase subunit 7A isoform mitochondrial | mitochondrial | R |  |  |
| 10 | GEY_27433 | cytochrome c oxidase subunit 6c | mitochondrial | R |  |  |
| 11 | GEY_11261 | cytochrome c oxidase subunit 4 isoform mitochondrial | mitochondrial | R | D |  |
| 12 | GEY_32484 | presenilins-associated rhomboid-like protein mitochondrial | mitochondrial | R | D | PS |
| 13 | GEY_18390 | dihydrolipoyllysine-residue succinyltransferase component of 2-oxoglutarate dehydrogenase mitochondrial-like protein | mitochondrial | R |  |  |
| 14 | GEY_25926 | Diablo homolog, mitochondrial | mitochondrial | R | D |  |
| 15 | GEY_30467 | mitochondrial coenzyme A transporter SLC25A42-like | mitochondrial | R | D | PS |
| 16 | GEY_06415 | transcription elongation mitochondrial-like | mitochondrial | R | D | PS |
| 17 | GEY_30482 | ATP synthase subunit delta,mitochondrial | mitochondrial | R | D |  |
| 18 | GEY_06998 | ATP synthase subunit gamma,mitochondrial | mitochondrial | R | D | PS |
| 19 | GEY_30910 | D-beta-hydroxybutyrate dehydrogenase, mitochondrial | mitochondrial | R |  |  |
| 20 | GEY_15158 | alpha-ketoglutarate-dependent dioxygenase alkB homolog 7, mitochondrial-like | mitochondrial | R | D |  |
| 21 | GEY_01546 | mitochondrial cardiolipin hydrolase-like isoform X1 | mitochondrial | R |  |  |
| 22 | GEY_26048 | 1,25-dihydroxyvitamin D(3) 24-hydroxylase, mitochondrial | mitochondrial | R | D | PS |
| 23 | GEY_13533 | cytochrome b-c1 complex subunit 7-like | mitochondrial | R |  |  |
| 24 | GEY_19983 | mitochondrial import inner membrane  translocase subunit TIM44-lik | mitochondrial | R |  |  |
| 25 | GEY_31024 | ERO1-like protein alpha-like | | R |  |  |
| 26 | GEY_26086 | glutathione transferase omega-1 | | R |  |  |
| 27 | GEY_14238 | glutathione s-transferase | | R |  |  |
| 28 | GEY_08735 | inactive hydroxysteroid dehydrogenase-like protein 1-like | | R |  |  |
| 29 | GEY_22565 | cytochrome c-type heme lyase isoform X1 | | R |  |  |
| 30 | GEY_31878 | synaptic vesicle membrane protein VAT-1 homolog | | R | D | PS |
| 31 | GEY_12118 | saccharopine dehydrogenase | | R | D | PS |
| 32 | GEY_27846 | metalloreductase STEAP4-like | | R | D |  |
| 33 | GEY_20265 | hydroxysteroid 11-beta-dehydrogenase 1-like protein-like | | R |  |  |
| 34 | GEY_16351 | FAD-dependent oxidoreductase domain-containing protein 1-like | | R |  |  |
| 35 | GEY_23235 | dehydrogenase reductase SDR family member 13-like | | R | D | PS |
| 36 | GEY_03675 | cholesterol desaturase daf-36-like | | R | D | PS |
| 37 | GEY_12202 | small RNA 2'-O-methyltransferase-like | | R | D |  |
| 38 | GEY_10737 | piwi-like protein 1 | | R | D | PS |
| 39 | GEY_20705 | H-2 class I histocompatibility L-D alpha chain-like | | R |  |  |
| 40 | GEY_26736 | rhodopsin kinase | | R |  |  |
| 41 | GEY_30326 | leukotriene-B(4) omega-hydroxylase 2-like | | R | D | PS |
| 42 | GEY_17335 | malate dehydrogenase, cytoplasmic | | R | D | PS |
| 43 | GEY_12821 | 3-hydroxyanthranilate 3,4-dioxygenase-like | | R | D |  |
| 44 | GEY_21692 | 3-oxo-5-alpha-steroid 4-dehydrogenase 1-like | | R | D |  |
| 45 | GEY_22407 | sulfiredoxin-1-like | | R |  |  |
| 46 | GEY_18585 | 3-hydroxy-3-methylglutaryl-coenzyme A reductase-like | | R | D | PS |

^1^R: rapidly evolving gene with *dN* in the upper 10% of *N. coriiceps*, ^2^D: *N. coriiceps* linege-specific gene in tree (*q* < 0.05), ^3^PS: gene evolving under positive selection (*q* < 0.05)

**Table S13. Sequencing reads used in analysis of RNA-Seq under stresses.**

| **Sample ID** | **Run Format** | **Max ReadLength** | **Assembled into genome** | **Trimmed reads** | **Total reads** |
| --- | --- | --- | --- | --- | --- |
| Blood Con 1 | 2x101 | 101bp | 27,628,526 | 32,868,066 | 37,978,348 |
| Blood Con 2 | 2x101 | 101bp | 21,406,390 | 26,896,420 | 33,777,442 |
| Blood Cold 2 | 2x101 | 101bp | 24,455,254 | 29,902,570 | 37,743,234 |
| Blood Cold 3 | 2x101 | 101bp | 24,387,552 | 28,241,390 | 32,592,560 |
| Blood Heat 1 | 2x101 | 101bp | 25,688,242 | 33,101,200 | 38,834,040 |
| Blood Heat 2 | 2x101 | 101bp | 22,968,974 | 27,745,734 | 34,832,290 |
| Brain Con 2 | 2x101 | 101bp | 28,751,704 | 34,523,836 | 39,975,118 |
| Brain Con 3 | 2x101 | 101bp | 22,690,202 | 26,920,302 | 33,616,256 |
| Brain Cold 2 | 2x101 | 101bp | 24,071,220 | 28,815,132 | 33,196,790 |
| Brain Cold 3 | 2x101 | 101bp | 18,051,382 | 21,672,420 | 26,898,786 |
| Brain Heat 1 | 2x101 | 101bp | 25,975,718 | 31,544,428 | 36,471,762 |
| Brain Heat 2 | 2x101 | 101bp | 23,208,080 | 27,996,790 | 34,812,674 |

**Table S14. The result of RNA-seq.** Detail gene list are shown in Table S16 - S22.

| **Tissue** | **Experiments** | **Up Regulation** | **Down Regulation** | **Total** |
| --- | --- | --- | --- | --- |
| Blood | Control vs Cold stress | 46 | 180 | 226 |
|  | Control vs Heat stress | 31 | 206 | 237 |
| Brain | Control vs Cold stress | 105 | 177 | 282 |
|  | Control vs Heat stress | 86 | 148 | 234 |

**Table S15. Upregulated genes in blood under both cold and heat stress.**

| **Locus tag** | **Gene^1^** | **Fold change (log2)** | |
| --- | --- | --- | --- |
|  |  | **Heat** | **Cold** |
| GEY_06579 | sam domain and hd domain-containing protein partial | 8.15528 | 6.04577 |
| GEY_05781 | endothelin-converting enzyme 2 | 7.48627 | 9.20282 |
| GEY_29601 | endothelin-converting enzyme 2-like | 6.76716 | 8.07018 |
| GEY_11487 | inosine-uridine preferring nucleoside hydrolase-like | 5.76777 | 4.56045 |
| GEY_28068 | lysophospholipid acyltransferase lpcat4-like | 5.30567 | 3.83440 |
| GEY_31044 | **heat shock protein ssb1** | 4.12787 | 4.42467 |
| GEY_31042 | **heat shock protein 70** | 3.89580 | 4.13861 |
| GEY_22197 | c-binding protein | 3.79243 | 3.49416 |
| GEY_24796 | sam domain and hd domain-containing protein 1-like | 2.94223 | 4.00708 |
| GEY_30844 | hypothetical protein | 2.77533 | 4.00278 |
| GEY_12222 | tenascin r ( janusin)-like | 2.74675 | 2.32422 |
| GEY_19772 | **heat shock protein 40** | 2.44724 | 2.30014 |
| GEY_08470 | guanine deaminase-like | 2.13688 | 3.34807 |

**^1^**Hsps related in HSR are presented with bold characters

**Table S16. GO enrichment test in blood under heat stress.**

| **GO term^1^** | **Ontology** | **Description** |  |  | **p-value** | **FDR** |
| --- | --- | --- | --- | --- | --- | --- |
| **GO:0006986** | P | response to unfolded protein |  |  | 1.2E-44 | 5.1E-43 |
| **GO:0051789** | P | response to protein stimulus |  |  | 1.1E-36 | 2.5E-35 |
| **GO:0009607** | P | response to biotic stimulus |  |  | 1.4E-23 | 2E-22 |
| **GO:0006950** | P | response to stress |  |  | 0.00000047 | 0.0000051 |
| GO:0044248 | P | cellular catabolic process |  |  | 0.00058 | 0.0051 |
| GO:0009056 | P | catabolic process |  |  | 0.0012 | 0.0074 |
| **GO:0050896** | P | response to stimulus |  |  | 0.0012 | 0.0074 |
| GO:0009143 | P | nucleoside triphosphate catabolic process |  |  | 0.003 | 0.0078 |
| GO:0009146 | P | purine nucleoside triphosphate catabolic process |  |  | 0.003 | 0.0078 |
| GO:0009207 | P | purine ribonucleoside triphosphate catabolic process |  |  | 0.003 | 0.0078 |
| GO:0009203 | P | ribonucleoside triphosphate catabolic process |  |  | 0.003 | 0.0078 |
| GO:0009154 | P | purine ribonucleotide catabolic process |  |  | 0.003 | 0.0078 |
| GO:0009261 | P | ribonucleotide catabolic process |  |  | 0.003 | 0.0078 |
| GO:0009117 | P | nucleotide metabolic process |  |  | 0.0019 | 0.0078 |
| **GO:0010033** | P | response to organic substance |  |  | 0.0018 | 0.0078 |
| GO:0055086 | P | nucleobase, nucleoside and nucleotide metabolic process |  |  | 0.003 | 0.0078 |
| GO:0006753 | P | nucleoside phosphate metabolic process |  |  | 0.0019 | 0.0078 |
| GO:0006195 | P | purine nucleotide catabolic process |  |  | 0.0034 | 0.0083 |
| GO:0009166 | P | nucleotide catabolic process |  |  | 0.0038 | 0.0088 |
| GO:0046700 | P | heterocycle catabolic process |  |  | 0.0054 | 0.012 |
| GO:0009141 | P | nucleoside triphosphate metabolic process |  |  | 0.0068 | 0.013 |
| GO:0009144 | P | purine nucleoside triphosphate metabolic process |  |  | 0.0067 | 0.013 |
| GO:0009199 | P | ribonucleoside triphosphate metabolic process |  |  | 0.0068 | 0.013 |
| GO:0009205 | P | purine ribonucleoside triphosphate metabolic process |  |  | 0.0067 | 0.013 |
| GO:0009150 | P | purine ribonucleotide metabolic process |  |  | 0.0095 | 0.017 |
| GO:0009259 | P | ribonucleotide metabolic process |  |  | 0.011 | 0.019 |
| GO:0006163 | P | purine nucleotide metabolic process |  |  | 0.023 | 0.037 |
| GO:0044281 | P | small molecule metabolic process |  |  | 0.025 | 0.039 |
| GO:0042221 | P | response to chemical stimulus |  |  | 0.031 | 0.047 |
| **GO:0051082** | F | unfolded protein binding |  |  | 1.1E-15 | 2.8E-14 |

^1^Shared GO terms under both cold stress and heat stress condition are presented with bold characters

**Table S17. GO enrichment test in blood under cold stress.**

| **GO term^1^** | **Ontology** | **Description** |  | **p-value** | **FDR** |
| --- | --- | --- | --- | --- | --- |
| **GO:0006986** | P | response to unfolded protein |  | 5.1E-30 | 3E-28 |
| **GO:0051789** | P | response to protein stimulus |  | 1.1E-24 | 3.3E-23 |
| GO:0009607 | P | response to biotic stimulus |  | 9E-16 | 1.7E-14 |
| **GO:0006457** | P | protein folding |  | 0.0000032 | 0.000046 |
| **GO:0006950** | P | response to stress |  | 0.000004 | 0.000046 |
| **GO:0010033** | P | response to organic substance |  | 0.00046 | 0.0044 |
| **GO:0050896** | P | response to stimulus |  | 0.00053 | 0.0044 |
| **GO:0051082** | F | unfolded protein binding |  | 1.1E-19 | 1.6E-18 |
| GO:0046983 | F | protein dimerization activity |  | 0.0019 | 0.013 |

**^1^**Shared GO terms under both cold stress and heat stress condition are presented with bold characters

**Table S18. Top blood-specific genes and their transcript percentages in whole blood transcriptomes.**

|  | **Locus_taq** | **gene** | **% of the total transcripts from whole blood** |
| --- | --- | --- | --- |
| 1 | GEY_14634 | hemoglobin beta 2 | 14.2% |
| 2 | GEY_14631 | hemoglobin alpha 2 | 6.9% |
| 3 | GEY_18816 | beta-2 microglobulin | 1.1% |
| 4 | GEY_21714 | ferritin high chain | 1.0% |
| 5 | GEY_01483 | band 3 anion exchange | 1.0% |
| 6 | GEY_12383 | hyaluronan and proteoglycan link protein 3 | 0.8% |
| 7 | GEY_28517 | mhc class ii antigen-associated invariant chain | 0.8% |
| 8 | GEY_14008 | transmembrane protein 205 | 0.6% |
| 9 | GEY_30148 | hypothetical protein | 0.6% |
| 10 | GEY_13157 | ribosomal protein l6 | 0.5% |
| 11 | GEY_19604 | 40s ribosomal protein s24 | 0.5% |
| 12 | GEY_18726 | 60s ribosomal protein l23a | 0.5% |
| 13 | GEY_16957 | carbonic anhydrase | 0.5% |
| 14 | GEY_29959 | stress protein hsc70-1 | 0.4% |
| 15 | GEY_12294 | 60s ribosomal protein l19 | 0.4% |
| 16 | GEY_03612 | 5-aminolevulinate erythroid protein | 0.4% |
| 17 | GEY_22589 | sushi domain-containing protein 1 | 0.4% |
| 18 | GEY_28123 | 40s ribosomal protein s26 | 0.4% |
| 19 | GEY_05071 | 60s ribosomal protein l32 | 0.4% |
| 20 | GEY_15898 | 60s ribosomal protein l7 | 0.4% |

**Table S19. Down-regulated genes in blood under cold stress.**

| **GO term^1^** | **Ontology** | **Description** |  |  | **p-value** | **FDR** |
| --- | --- | --- | --- | --- | --- | --- |
| **GO:0003779** | **F** | **actin binding** |  |  | 0.00011 | 0.003 |
| **GO:0020037** | **F** | **heme binding** |  |  | 0.00017 | 0.003 |
| **GO:0046906** | **F** | **tetrapyrrole binding** |  |  | 0.00027 | 0.0032 |
| **GO:0008092** | **F** | **cytoskeletal protein binding** |  |  | 0.00042 | 0.0038 |
| **GO:0005506** | **F** | **iron ion binding** |  |  | 0.0018 | 0.013 |
| GO:0070011 | F | peptidase activity, acting on L-amino acid peptides |  |  | 0.0051 | 0.031 |
| GO:0043169 | F | cation binding |  |  | 0.0085 | 0.032 |
| GO:0008233 | F | peptidase activity |  |  | 0.0089 | 0.032 |
| GO:0043167 | F | ion binding |  |  | 0.0087 | 0.032 |
| GO:0046872 | F | metal ion binding |  |  | 0.0071 | 0.032 |

**^1^**Shared GO terms under both cold stress and heat stress condition are presented with bold characters

**Table S20. Downregulated genes in blood under heat stress.**

| **GO term^1^** | **Ontology** | **Description** |  |  | **p-value** | **FDR** |
| --- | --- | --- | --- | --- | --- | --- |
| GO:0032535 | P | regulation of cellular component size |  |  | 0.0000021 | 0.00013 |
| GO:0090066 | P | regulation of anatomical structure size |  |  | 0.000011 | 0.00034 |
| GO:0016049 | P | cell growth |  |  | 0.00026 | 0.0053 |
| GO:0008361 | P | regulation of cell size |  |  | 0.00037 | 0.0056 |
| **GO:0020037** | **F** | **heme binding** |  |  | 5.2E-10 | 0.000000023 |
| **GO:0046906** | **F** | **tetrapyrrole binding** |  |  | 5.9E-09 | 0.00000013 |
| **GO:0005506** | **F** | **iron ion binding** |  |  | 0.000011 | 0.00016 |
| **GO:0003779** | **F** | **actin binding** |  |  | 0.00027 | 0.003 |
| GO:0005085 | F | guanyl-nucleotide exchange factor activity |  |  | 0.0034 | 0.025 |
| **GO:0008092** | **F** | **cytoskeletal protein binding** |  |  | 0.0029 | 0.025 |

**^1^**Shared GO terms under both cold stress and heat stress condition are presented with bold characters

**Table S21. Downregulated genes in blood under both cold and heat stress.**

| **Locus tag** | **Gene** | **Fold change (log2)** | |
| --- | --- | --- | --- |
|  |  | **Heat** | **Cold** |
| GEY_04391 | PREDICTED: hypothetical protein LOC100705382 | -1.83254 | -1.9221 |
| GEY_24135 | PREDICTED: myosin-9-like | -2.02168 | -1.96188 |
| GEY_15356 | actin-related protein 2 3 complex subunit 1b-like | -1.98999 | -1.97255 |
| GEY_10076 | neuronal nitric oxide synthase | -6.56712 | -3.39698 |
| GEY_31431 | pleckstrin homology sec7 and coiled-coil domains-binding protein | -6.22671 | -4.05701 |
| GEY_18730 | ---NA--- | -6.1307 | -3.96349 |
| GEY_17545 | c-c chemokine receptor type 2-like | -6.10662 | -3.93707 |
| GEY_16698 | probable tumor suppressor protein mn1 | -5.92829 | -3.75834 |
| GEY_07115 | bactericidal permeability-increasing protein | -5.8471 | -4.41522 |
| GEY_32352 | cd209 antigen-like | -5.74014 | -6.15595 |
| GEY_03512 | loc733422 protein | -5.59842 | -3.20676 |
| GEY_23074 | c-x-c chemokine receptor type 5-like | -5.51174 | -4.19058 |
| GEY_17563 | neural cell adhesion molecule 1-like | -5.24571 | -4.98282 |
| GEY_31921 | slit homolog 1 | -5.16722 | -3.16702 |
| GEY_15033 | sh2 domain-containing protein 3c-like | -5.0852 | -3.17842 |
| GEY_21306 | c-type lectin a | -5.05457 | -3.81764 |
| GEY_21755 | b-cell receptor cd22 precursor | -4.99296 | -4.22162 |
| GEY_10031 | proactivator polypeptide-like | -4.97501 | -3.9805 |
| GEY_11678 | cd81 antigen-like | -4.96388 | -2.57192 |
| GEY_28138 | nad (+)--arginine adp-ribosyltransferase 2-like | -4.94426 | -5.09755 |
| GEY_02813 | cathepsin l-like | -4.93305 | -5.08506 |
| GEY_06662 | type i cytoskeletal 18-like | -4.89286 | -2.72326 |
| GEY_06044 | junctional adhesion molecule b-like | -4.88152 | -4.7125 |
| GEY_02494 | phosphatidylinositol -trisphosphate 3-phosphatase tpte2-like | -4.87446 | -2.7662 |
| GEY_19148 | pou domain class 2-associating factor 1 | -4.73735 | -4.73933 |
| GEY_31937 | PREDICTED: uncharacterized protein LOC101163879 | -4.72546 | -2.60442 |
| GEY_16084 | 15-hydroxyprostaglandin dehydrogenase | -4.70224 | -4.12024 |
| GEY_09695 | b-cell antigen receptor complex-associated protein beta chain-like | -4.67893 | -4.09498 |
| GEY_14681 | interferon regulatory factor 4-like | -4.66052 | -4.24546 |
| GEY_09077 | proactivator polypeptide-like | -4.62646 | -3.80577 |
| GEY_28632 | endothelin b receptor-like | -4.5759 | -2.63733 |
| GEY_31184 | neuropilin 1a | -4.48935 | -3.48929 |
| GEY_08714 | mast cell protease 2-like | -4.43055 | -6.6543 |
| GEY_02416 | inositol-trisphosphate 3-kinase a-like | -4.42416 | -3.83941 |
| GEY_27847 | serum amyloid p-component precursor | -4.3796 | -3.53408 |
| GEY_18285 | p2y purinoceptor 12-like | -4.28293 | -2.63903 |
| GEY_18014 | cd9 antigen | -4.18886 | -4.60492 |
| GEY_16650 | progranulin type i | -4.17238 | -3.39552 |
| GEY_06042 | ---NA--- | -4.09978 | -3.89456 |
| GEY_20160 | inverted formin-2-like | -4.08488 | -2.6201 |
| GEY_21836 | cathepsin h precursor | -4.07283 | -3.10149 |
| GEY_07198 | leucine-rich repeat-containing protein 33-like | -4.0567 | -4.05686 |
| GEY_30801 | neuron navigator 1 | -4.04501 | -4.3529 |
| GEY_12802 | thrombopoietin receptor precursor | -4.03733 | -2.45252 |
| GEY_17058 | ras-specific guanine nucleotide-releasing factor 2 | -4.01341 | -4.01402 |
| GEY_25491 | p2y purinoceptor 10-like | -3.92839 | -3.53613 |
| GEY_32112 | immunoglobulin heavy chain variable region | -3.92574 | -4.07927 |
| GEY_18232 | cordon-bleu 1-like | -3.92362 | -4.66062 |
| GEY_15162 | af437741_1 heavy chain secretory form | -3.92255 | -2.07544 |
| GEY_24787 | lanosterol 14-alpha demethylase-like | -3.90703 | -2.77128 |
| GEY_29064 | adenosine deaminase domain-containing protein 2-like | -3.83084 | -5.24613 |
| GEY_13722 | b- and t-lymphocyte attenuator-like | -3.82681 | -4.56478 |
| GEY_32240 | b-cell antigen receptor complex-associated protein alpha chain-like | -3.77796 | -3.53972 |
| GEY_04716 | protein nlrc3-like | -3.74646 | -3.16146 |
| GEY_14922 | thyroid hormone receptor-associated protein 3 | -3.69841 | -2.83808 |
| GEY_27006 | monoglyceride lipase | -3.69774 | -4.33497 |
| GEY_03639 | integrin beta-3-like | -3.69494 | -3.29659 |
| GEY_01588 | filamin-a-like isoform 2 | -3.66029 | -3.15409 |
| GEY_23606 | protein-glutamine gamma-glutamyltransferase k-like | -3.65004 | -4.28774 |
| GEY_27739 | placenta-specific gene 8 | -3.64578 | -2.66706 |
| GEY_11900 | protein s100-a1-like | -3.63968 | -3.05719 |
| GEY_18160 | disabled homolog 2-interacting | -3.62609 | -3.58856 |
| GEY_15167 | immunoglobulin delta heavy chain | -3.62408 | -4.06303 |
| GEY_26561 | leucine-rich repeat-containing protein 32-like | -3.61868 | -2.86877 |
| GEY_14136 | nuclear factor ovary-like | -3.58532 | -4.41734 |
| GEY_00829 | myelin-associated glyco | -3.54995 | -5.18736 |
| GEY_13365 | prostaglandin g h synthase 1-like | -3.49773 | -3.91298 |
| GEY_04743 | 15-hydroxyprostaglandin dehydrogenase | -3.46137 | -5.87947 |
| GEY_00783 | annexin max3 | -3.45512 | -2.20892 |
| GEY_03045 | kelch-like protein 13-like | -3.43341 | -3.77499 |
| GEY_25496 | sh3 domain-binding glutamic acid-rich-like protein | -3.40878 | -3.15883 |
| GEY_22637 | ---NA--- | -3.33125 | -3.16116 |
| GEY_20964 | cytokine receptor common subunit beta-like | -3.32005 | -4.60995 |
| GEY_11730 | transcription factor -like | -3.30445 | -3.92939 |
| GEY_16553 | glypican 1 precursor | -3.29513 | -4.29504 |
| GEY_31713 | phosphatidylinositol -trisphosphate 5-phosphatase 2b-like | -3.29022 | -2.95669 |
| GEY_10312 | friend leukemia integration 1 transcription factor | -3.25582 | -3.25598 |
| GEY_30782 | ---NA--- | -3.23886 | -3.01775 |
| GEY_28285 | fh1 fh2 domain-containing protein 3-like | -3.22503 | -3.29897 |
| GEY_31426 | death-associated 1-a-like | -3.2103 | -2.14022 |
| GEY_20161 | adenylosuccinate synthetase isozyme 1 c-like | -3.14519 | -3.0462 |
| GEY_15085 | receptor-type tyrosine-protein phosphatase eta-like | -3.13659 | -2.43896 |
| GEY_01011 | filamin-a-like isoform 2 | -3.12333 | -2.70811 |
| GEY_00971 | matrix metalloproteinase-25-like | -3.1148 | -4.0928 |
| GEY_07238 | sodium potassium-transporting atpase subunit beta-2-like | -3.11293 | -2.97534 |
| GEY_12894 | vertebrate transmembrane 4 superfamily-like | -3.08623 | -4.23907 |
| GEY_23846 | dedicator of cytokinesis protein 2-like | -3.06672 | -2.83557 |
| GEY_06100 | amy-1-associating protein expressed in testis 1-like | -3.01894 | -3.19972 |
| GEY_26438 | denn domain-containing protein 5a | -3.00592 | -2.81311 |
| GEY_27164 | capping protein (actin filament) gelsolin-like | -2.99159 | -3.10774 |
| GEY_16873 | lymphoid-restricted membrane protein maternal long form | -2.98934 | -3.79696 |
| GEY_12806 | protein cyr61-like | -2.89961 | -2.83427 |
| GEY_21662 | guanine nucleotide-binding protein g subunit alpha-2-like | -2.88776 | -2.71846 |
| GEY_04631 | ras-related protein m-ras-like | -2.85555 | -2.85554 |
| GEY_04343 | low quality protein: myosin-11 | -2.8521 | -2.05446 |
| GEY_23295 | sam and sh3 domain-containing protein 3-like | -2.77696 | -3.19229 |
| GEY_19395 | creatine testis isozyme | -2.76187 | -2.69679 |
| GEY_19374 | phosphatidylinositol -trisphosphate 5-phosphatase 1-like | -2.73041 | -2.48083 |
| GEY_27217 | cysteine-rich protein 1 | -2.70219 | -3.12016 |
| GEY_01109 | tumor necrosis factor ligand superfamily member 13b-like | -2.69366 | -2.36561 |
| GEY_23211 | leukocyte surface antigen cd53 | -2.69208 | -2.6035 |
| GEY_32142 | coagulation factor xiii a chain | -2.62851 | -2.05728 |
| GEY_04586 | synapse differentiation-inducing gene protein 1-like | -2.60554 | -3.07998 |
| GEY_03320 | zgc:162304 protein | -2.5339 | -3.61898 |
| GEY_10712 | lim domain-containing protein 2-like | -2.45282 | -2.57417 |
| GEY_19128 | cytochrome b558 alpha-subunit | -2.41629 | -3.25878 |
| GEY_14103 | egf-like module-containing mucin-like hormone receptor-like 1-like | -2.36468 | -2.74728 |
| GEY_12911 | megalocytivirus-induced protein 1 | -2.35763 | -2.44392 |
| GEY_20328 | PREDICTED: moesin-like | -2.2046 | -2.62952 |
| GEY_10658 | PREDICTED: coronin-1A-like | -2.12504 | -2.89972 |
| GEY_08429 | krueppel-like factor 2-like | -1.99358 | -2.21075 |
| GEY_18384 | matrix metalloproteinase-9 precursor | -1.95839 | -4.04817 |

**Table S22. Shared genes in downregulated group under heat and cold stress in the brain.**

|  | **Locus taq** | **Gene** | **Fold change in cold** | **p-value** | **Fold change in heat** | **p-value** | **In hypoxia** |
| --- | --- | --- | --- | --- | --- | --- | --- |
| 1 | GEY_26613 | protein cornichon homolog 2 | -6.10336 | 0.00732137 | -3.74311 | 0.0327655 | + |
| 2 | GEY_11826 | sarcoplasmic endoplasmic reticulum calcium atpase 2-like protein | -2.90571 | 0.00706631 | -3.15203 | 0.00733782 |  |
| 3 | GEY_22240 | cocaine- and amphetamine-regulated transcript | -4.38574 | 0.000723486 | -3.78289 | 0.0129315 | + |
| 4 | GEY_29894 | cgmp-specific 3 -cyclic phosphodiesterase-like protein | -3.68775 | 0.000161136 | -4.44665 | 3.79988E-05 |  |
| 5 | GEY_20176 | PREDICTED: opsin-5-like protein | -4.51221 | 0.0029093 | -5.73684 | 0.00212647 |  |
| 6 | GEY_20769 | unnamed protein product (syntaxin-1A-like) | -3.59546 | 0.0037893 | -3.73266 | 0.0198134 | + |
| 7 | GEY_25692 | glucagon family neuropeptides-like protein | -2.86953 | 0.038564 | -3.17225 | 0.0326696 |  |
| 8 | GEY_20590 | cocaine- and amphetamine-regulated transcript | -6.21846 | 8.82471E-06 | -5.44361 | 4.28593E-05 | + |
| 9 | GEY_15195 | pituitary adenylate cyclase-activating polypeptide type i receptor-like protein | -3.77615 | 0.000671423 | -3.66371 | 0.00275843 | + |
| 10 | GEY_24480 | neuropeptide b precursor | -2.3551 | 0.111826 | -3.90171 | 0.00281699 | + |
| 11 | GEY_31706 | sodium calcium exchanger 1-like protein | -4.72879 | 7.30066E-06 | -4.00589 | 0.000456884 | + |
| 12 | GEY_31708 | sodium calcium exchanger 1-like protein | -5.02039 | 1.25842E-07 | -4.21102 | 7.12194E-05 | + |
| 13 | GEY_09968 | cannabinoid receptor type 1b-like protein | -3.35365 | 0.02 | -4.09281 | 0.00687152 |  |
| 14 | GEY_32082 | sodium-driven chloride bicarbonate exchanger-like protein | -3.16397 | 0.0243106 | -5.50405 | 0.000778925 |  |
| 15 | GEY_18501 | vesicular glutamate transporter 2-like protein | -4.682 | 1.25842E-07 | -4.25708 | 3.38046E-05 |  |
| 16 | GEY_29155 | isotocin receptor-like protein | -2.78655 | 0.0614428 | -3.42618 | 0.0257937 | + |
| 17 | GEY_17515 | camk2a protein | -6.20101 | 4.89793E-08 | -5.10388 | 6.57316E-06 | + |
| 18 | GEY_26917 | muscarinic acetylcholine receptor m2-like protein | -3.17459 | 0.0506308 | -3.81422 | 0.0268612 | + |
| 19 | GEY_07853 | vesicular glutamate transporter 2-like protein | -3.91389 | 6.32626E-05 | -4.22594 | 9.46065E-05 |  |
| 20 | GEY_22275 | prepromelanin concentrating hormone | -12.9749 | 0 | -10.1997 | 0 | + |
| 21 | GEY_04957 | metabotropic glutamate receptor 5-like protein | -4.53192 | 0.000028677 | -4.05604 | 0.000774628 | + |
| 22 | GEY_24878 | g-protein coupled receptor 6-like protein | -5.55508 | 0.0243106 | -5.77969 | 0.0296731 |  |
| 23 | GEY_23950 | glucagon receptor-like protein | -3.42843 | 0.0545029 | -4.97496 | 0.0181517 |  |
| 24 | GEY_08729 | cannabinoid receptor type 1a-like protein | -3.33718 | 0.00118466 | -3.18561 | 0.00687152 | + |
