# Supplementary material for: The genome sequence of the Antarctic bullhead notothen reveals evolutionary adaptations to a cold environment
Source: Genome Biol. 2014 Sep 25;15(9):468. doi: 10.1186/s13059-014-0468-1 (PMC4192396; doi:10.1186/s13059-014-0468-1)
Supplement: Additional file 2: Figure S1. — Contig characteristics. (A) The contig length distribution shows that small size contig were incorporated into other contig. (B) Contig coverage. Figure S2: Comparison of the assembled genome with six BACs sequences. NUCmer alignments of the Celera genome assembly scaffolds (x-axis) and BAC sequences (y-axis) ordered and oriented such that the largest hits cluster. Figure S3: Gene ontology (GO) distribution after BlastX analysis for N. coriiceps transcriptome sequences as grouped by biological process (red), cellular component (cyan), and molecular function (grey). Figure S4: Cladogram representing phylogenetic relationship between selected 5,039 orthologous genes of six fishes. Figure S5: Sequence alignment of heat shock factor-1. The sites required for post-translational modification were well-conserved except Ser303, corresponding to human HSF1 in PDSM in Antarctic fish (N. coriiceps, Dragon fish and Icefish). Figure S6: Significantly over-represented GO terms under heat and cold stress among upregulated genes from blood. Figure S7: Tissue-specific expression of genes related to the HSR. (A) HSR and UPR were confirmed to be downregulated with RNA-Seq analysis in whole blood sample. (B) FKBP, HSP70, and HSP40 genes were confirmed to be downregulated in blood samples more than other tissues in HSR. XBP1 gene, the representative transcription factor in UPR, was also downregulated in blood sample. Figure S8: Significantly over-represented GO terms under heat and cold stress among downregulated genes from blood and gene expression included in heme binding (GO:0020037). (A,B) Significantly over-represented GO terms among the downregulated genes from blood showed the similar GO terms hierarchy. Five GO terms were shared in both cold and heat stress. (C) Results of RNA-Seq studies show the expression-pattern of genes included in GO:00200037. Figure S9: Toxicity of nitric oxide and superoxide. Figure S10: Forty-one GO terms focused on seven GO terms in the gene ontology h [file 13059_2014_468_MOESM2_ESM.docx]

**Additional file 2. Figure S1 – Figure S10.**


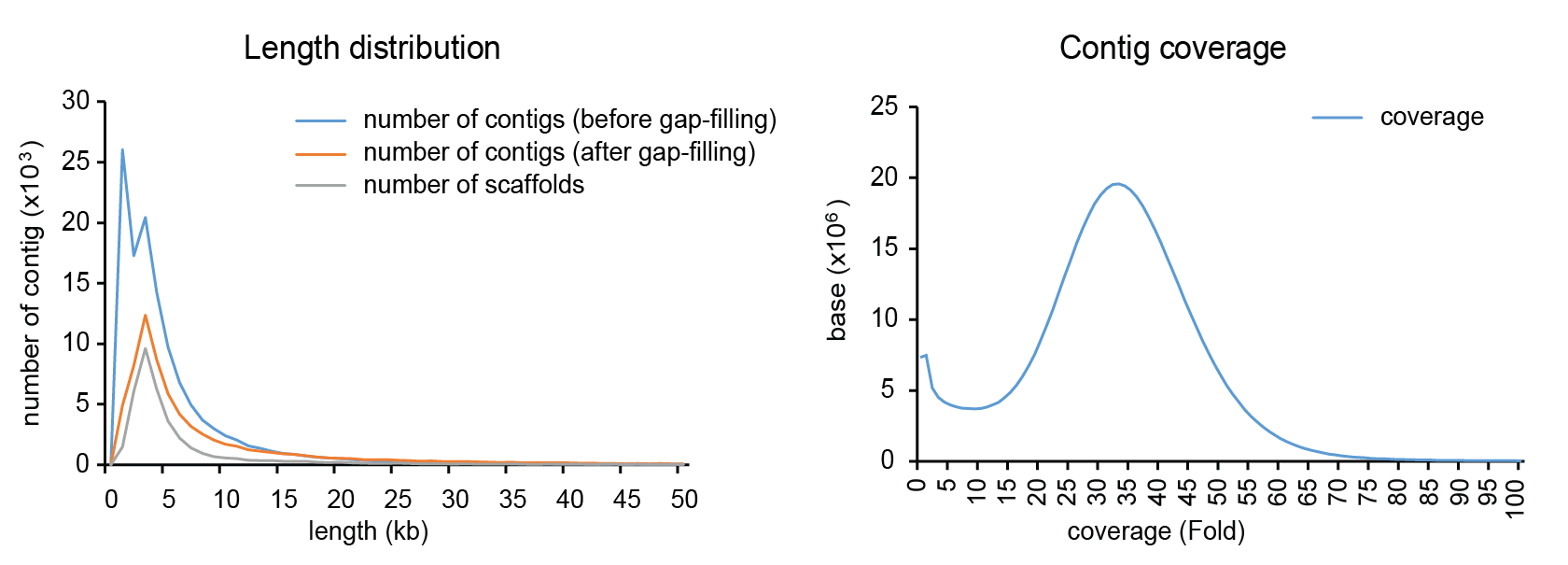


**A B**

**Figure S1. Contig characteristics. A**, The contig length distribution shows that small size contig were incorporated into other contig. **B,** Contig coverage.


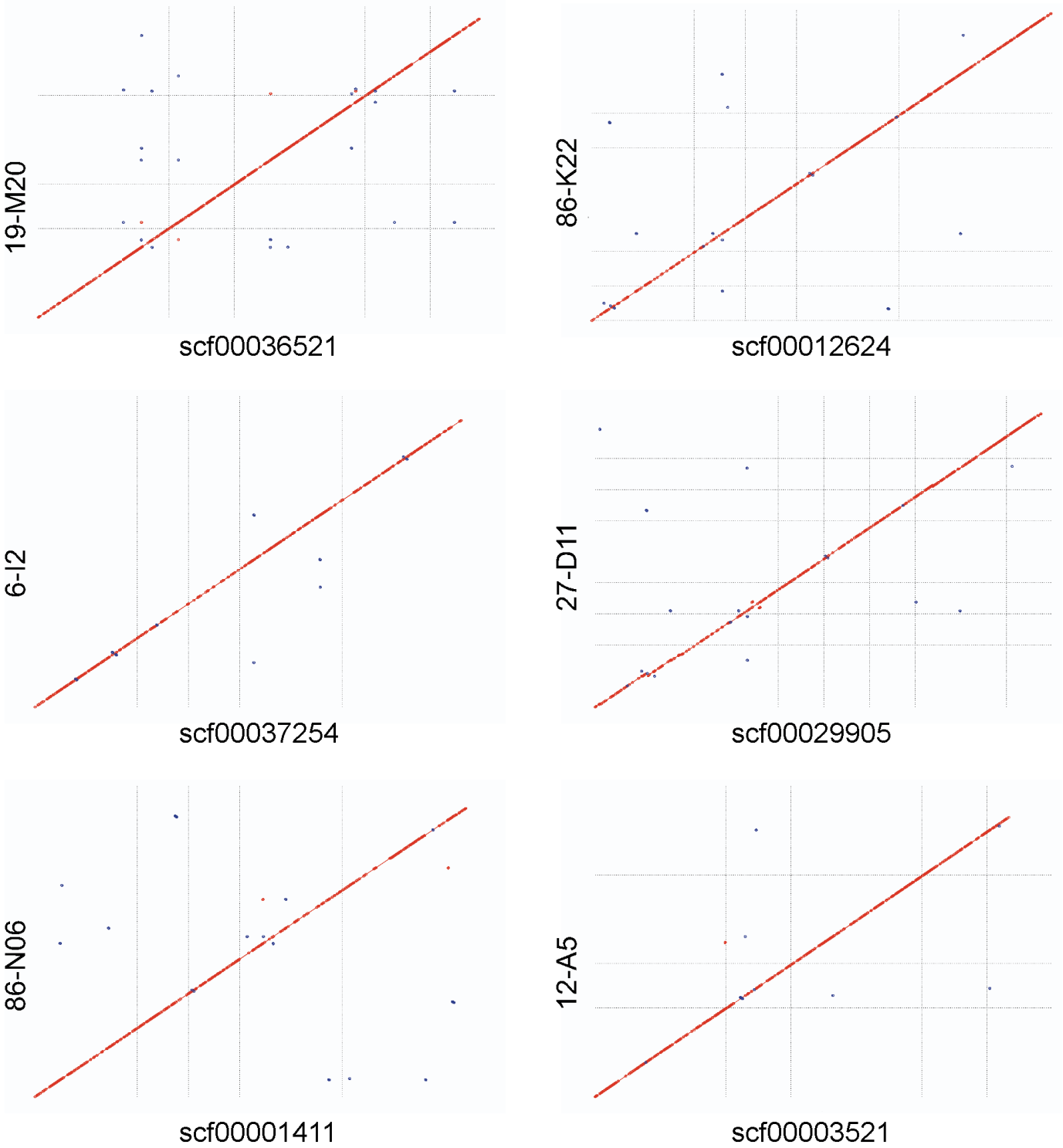


**Figure S2. Comparison of the assembled genome with 6 BACs sequences.** NUCmer alignments of the Celera genome assembly scaffolds (x-axis) and BAC sequences (y-axis) ordered and oriented such that the largest hits cluster.


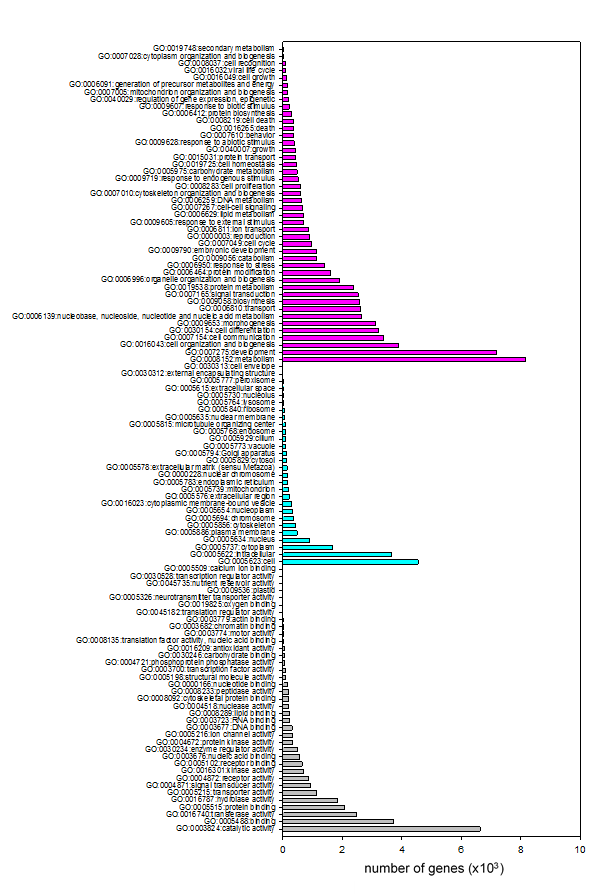


**Figure S3. Gene ontology (GO) distribution after BlastX analysis for N. coriiceps transcriptome sequences as grouped by biological process (red), cellular component (cyan) and molecular function (grey).**

**Figure S4. Cladogram representing phylogenetic relationship between selected 5,039 orthologous genes of six fishes.**

**
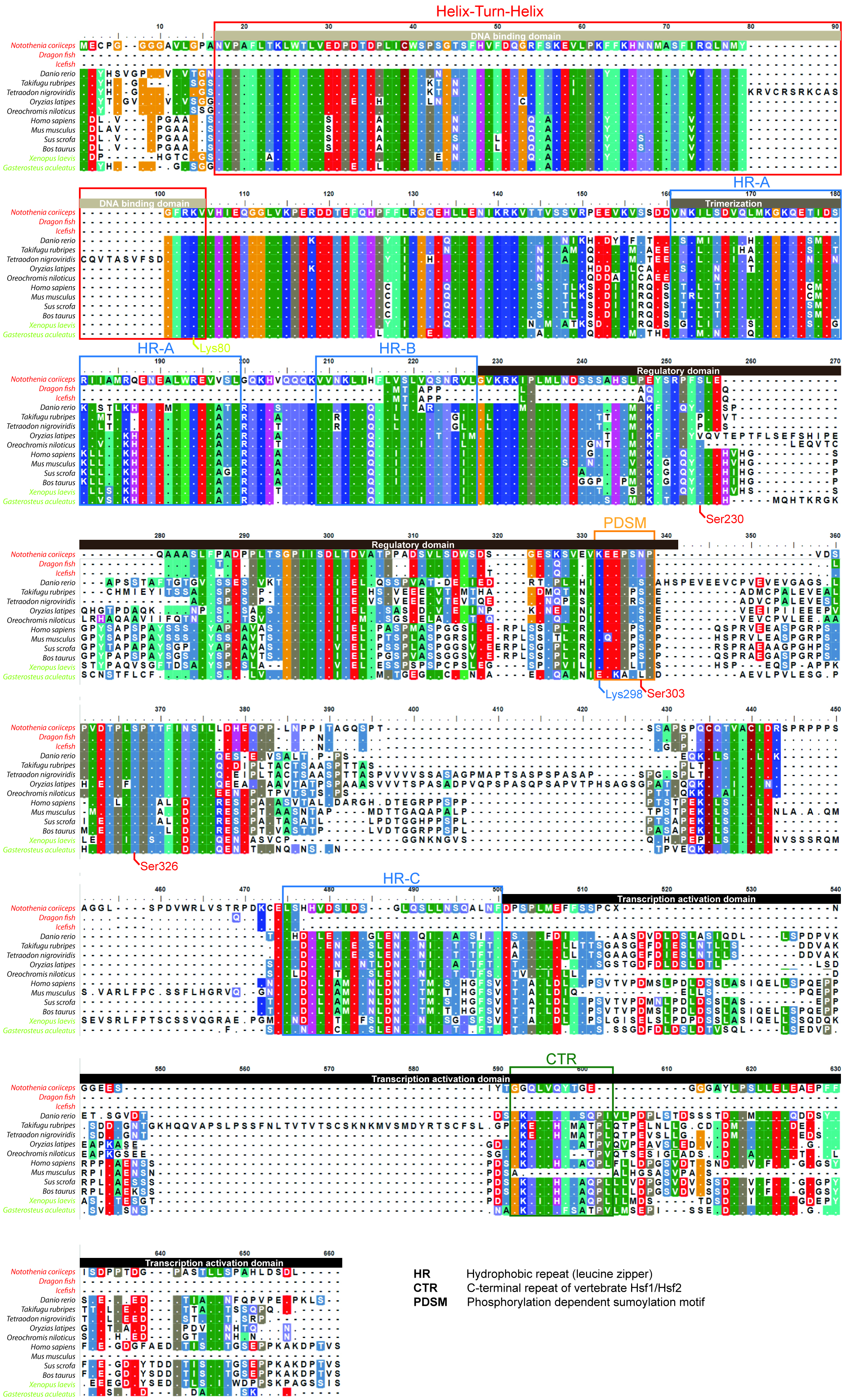
**

**Figure S5. Sequence alignment of heat shock factor-1.** The sites required for post-translational modification were well-conserved except Ser303, corresponding to human *HSF1* in PDSM in Antarctic fish (*N. coriiceps*, Dragon fish and Icefish).


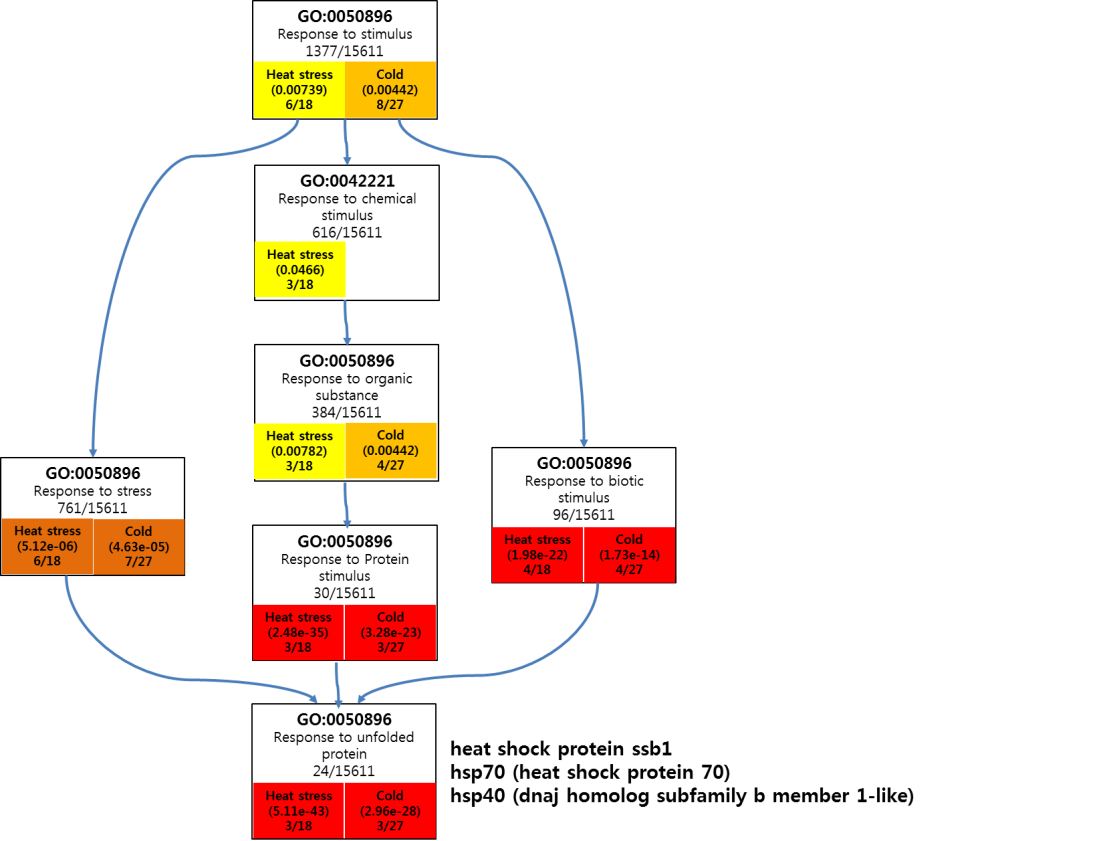


**Figure S6. Significantly overrepresented GO terms under heat and cold stress among upregulated genes from blood.**

**A**


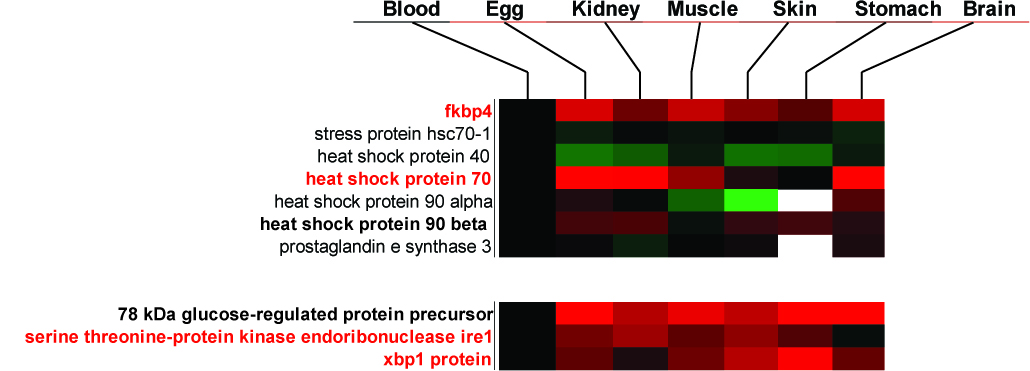


**B**


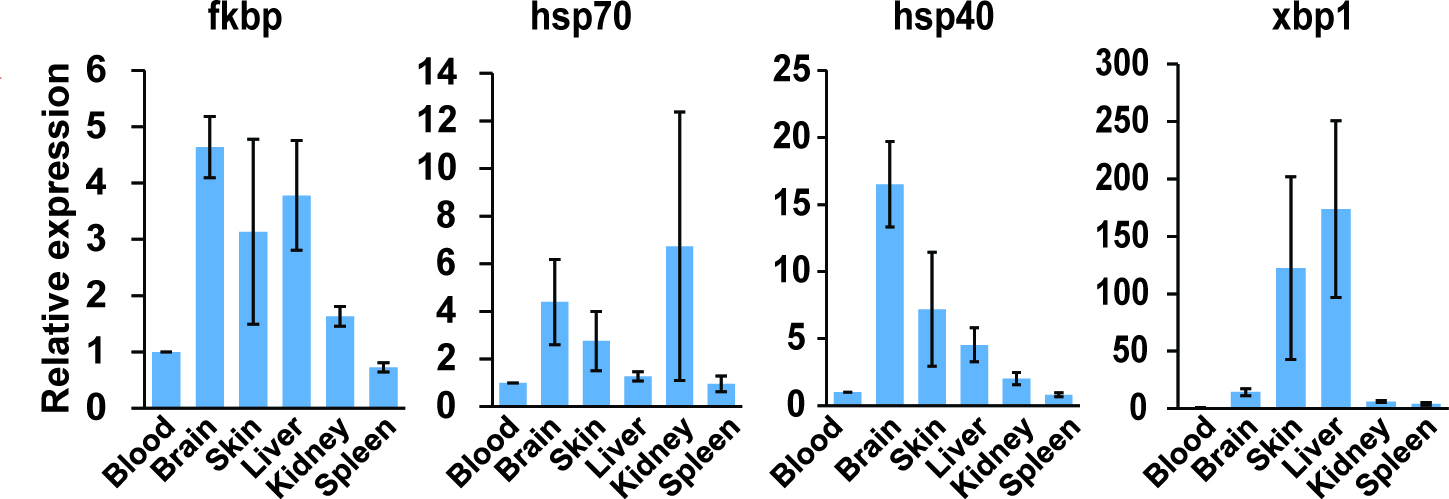


**Figure S7. Tissue-specific expression of genes related to the HSR. A,** HSR and UPR were confirmed to be downregulated with RNA-Seq analysis in whole blood sample. **B,** *FKBP*, *HSP70*, and *HSP40* genes were confirmed to be downregulated in blood samples more than other tissues in HSR. *XBP1* gene, the representative transcription factor in UPR, was also downregulated in blood sample.

**A B**


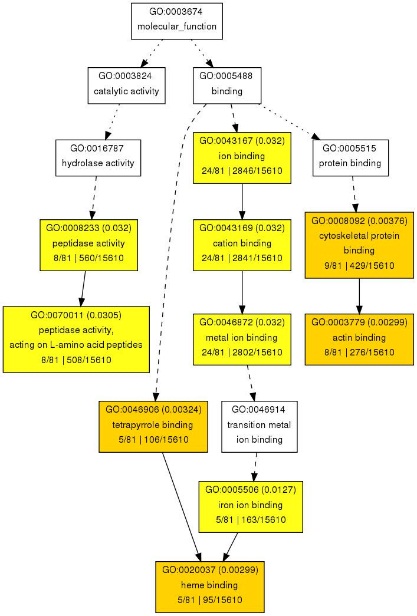

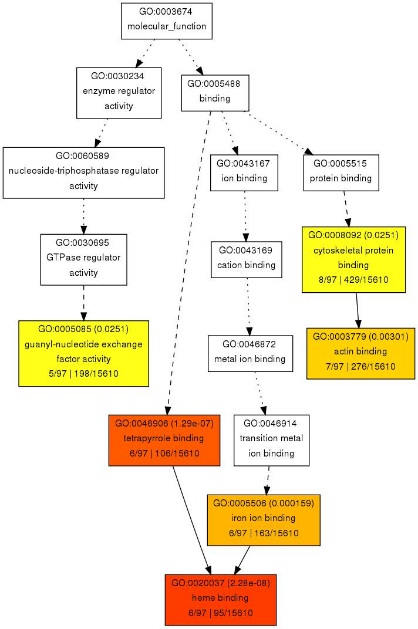


Cold stress Heat stress

**C**


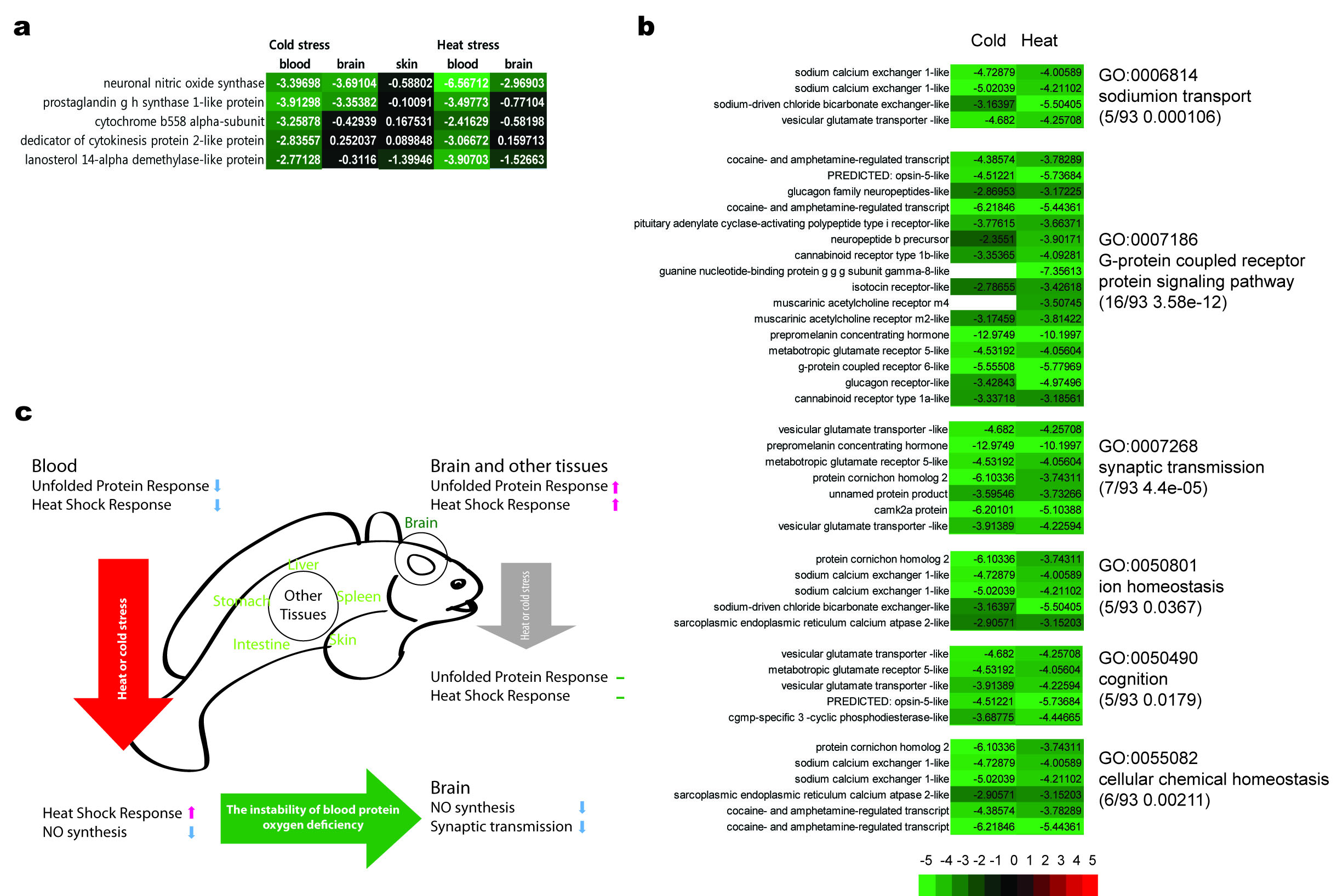


**Figure S8. Significantly overrepresented GO terms under heat and cold stress among downregulated genes from blood and gene expression included in heme binding (GO:0020037). A** and **B,** Significantly overrepresented GO terms among the downregulated genes from blood showed the similar GO terms hierarchy. 5 GO terms were shared in both cold and heat stress. **C,** Results of RNA-Seq studies show the expression-pattern of genes included in GO:00200037.


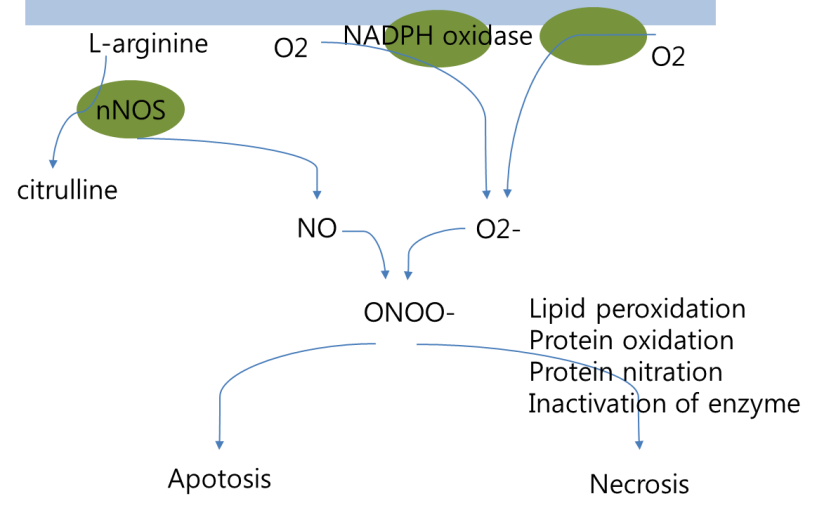


**Figure S9. Toxicity of nitric oxide and superoxide.**


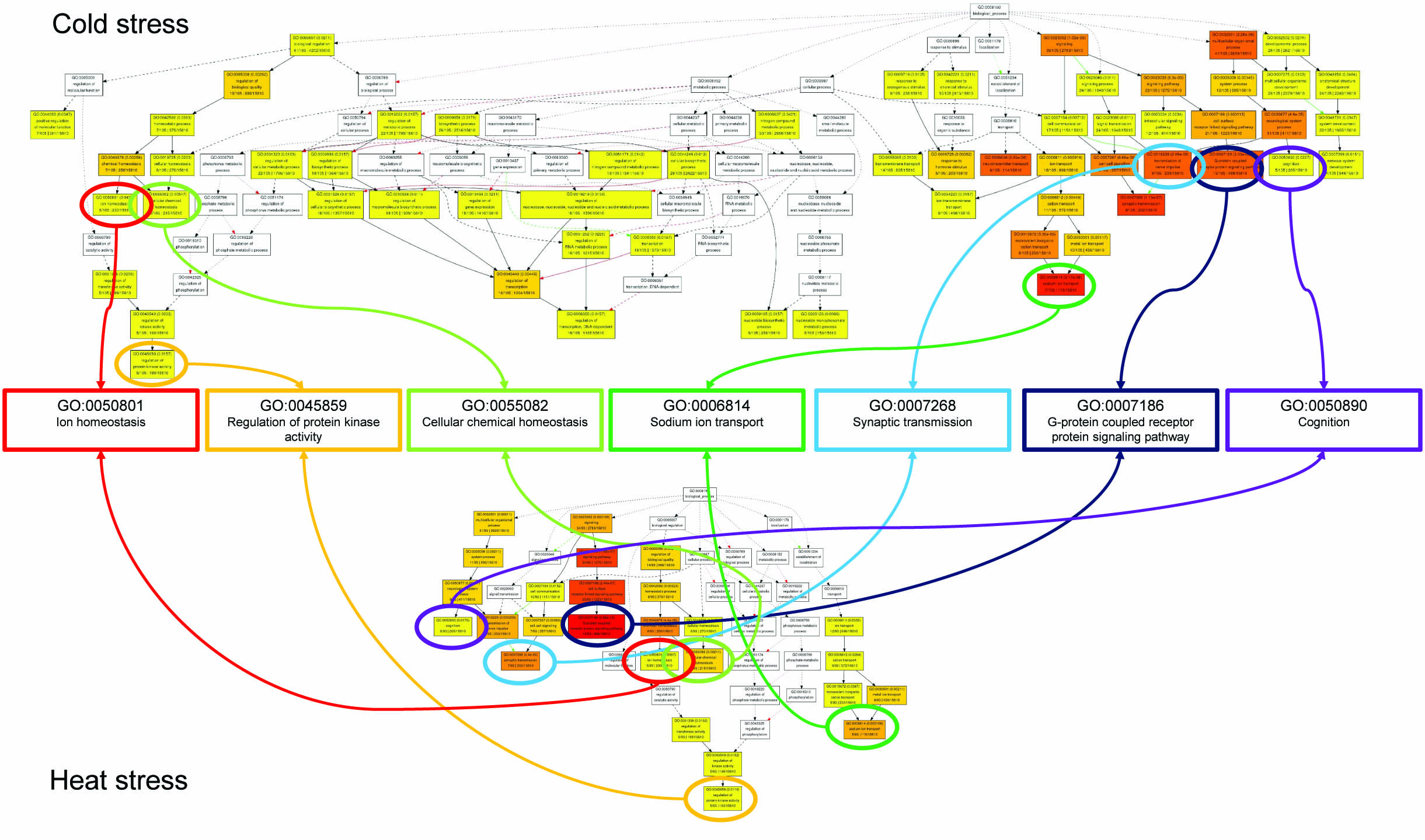


**Figure S10. 41 GO terms focused on 7 GO terms in the gene ontology hierarchy.**
