# Supplementary material for: The genome sequence of the Antarctic bullhead notothen reveals evolutionary adaptations to a cold environment
Source: Genome Biol. 2014 Sep 25;15(9):468. doi: 10.1186/s13059-014-0468-1 (PMC4192396; doi:10.1186/s13059-014-0468-1)
Supplement: Additional file 5: — Gene expression under temperature stress. [file 13059_2014_468_MOESM5_ESM.docx]

**Additional file 5.**

**Gene expression under temperature stress**

In RNA-seq experiments to the challenge of temperature stress, 112 were downregulated under both stresses (179 genes under cold and 206 genes under heat stress) and 5 GO terms were shared (10 GO terms for cold stress) (Molecular function, GO:0003779, actin binding; GO:0020037, heme binding; GO:0046906, tetrapyrrole binding; GO:0008092, cytoskeletal protein binding; and GO:0005506, iron ion binding) (Additional file 1: Table S19-S21 and Additional file 2: Fig. S8). We explored heme binding (GO:0020037), including genes related to NO synthesis and superoxides (Additional file 2: Fig. S9). Cytochrome b558 alpha-subunit is a component of Flavocytochrome b558. Heterodimers consist of the large glycoprotein gp91phox (phox forphagocyte oxidase) (beta subunit) and the small protein p22phox (alpha subunit), Flavocytochrome b558, which is anchored in the plasma membrane and functions as the catalytic core of NADPH oxidase to form superoxide through the transfer of electrons from NADPH to molecular oxygen [1, 2]. Neuronal nitric oxide synthase catalyzes the production of nitric oxide (NO) and superoxide from L-arginine in both the central and peripheral nervous system [3-6]. These enzymes are activated through hypoxic signaling in the brain and lung [7, 8]. The reactions of nitric oxide with superoxide generated from stress produces powerful and toxic oxidant peroxynitrite (ONOO^-^) [9, 10]. Next, peroxynitrite reacts relatively slowly with most biological molecules (lipids, DNA, and proteins), which damages the cell through lipid peroxidation, protein oxidation, protein nitration, and enzyme inactivation, leading to cell death by necrosis or apoptosis [4]. However, the expression of cytochrome b558 alpha-subunit and neuronal nitric oxide synthase in the blood and brain decreased after both heat and cold stress.

In the brain, 105 genes were upregulated under cold stress and 86 genes under heat stress. Based on a GO enrichment test with genes upregulated more than twofold under heat and cold, 13 GO terms were enriched under heat stress. A total of 177 genes were downregulated under cold stress, 148 genes were downregulated under heat stress, and 91 GO terms and 42 GO terms were significantly enriched (*p* < 0.05), respectively. A total of 94 genes and 41 GO terms were shared. Of the 41 shared GO terms, we further explored 7 GO terms including 23 genes in the gene ontology hierarchy (Additional file 2: Fig. S10). The majority of genes increased their expression under hypoxia or ischemia (Additional file 1: Table S22). These genes were not regulated by the heat shock response. However, the majority of genes were downregulated under heat or cold stresses. Hif1-regulated protein levels were constant under this stress based on the mRNA level [11]; however, we did not monitor HIF-1 protein level. The Muscarinic acetylcholine receptor (which inhibits the degradation of HIF-1) was downregulated [11, 12]. Other genes that damage the cell under hypoxia were confirmed to be downregulated, including the protein cornichon homolog 2 (the auxiliary molecule of AMPA receptor) and metabotropic glutamate receptor 5-like protein [13-15]. Under hypoxia, expression of leptin is known to be increased [16], and genes regulated by leptin were also upregulated under heat and cold stresses, including prepromelanin concentrating hormone and cocaine and amphetamine-regulated transcript [17, 18]. This may be how *N. coriiceps* limits damage from these stresses (such as hypoxia).

**Reference**

1. Koshkin V, Pick E: **Generation of superoxide by purified and relipidated cytochrome b559 in the absence of cytosolic activators**. *FEBS Lett* 1993, **327**(1):57-62.

2. Koshkin V, Pick E: **Superoxide production by cytochrome b559. Mechanism of cytosol-independent activation**. *FEBS Lett* 1994, **338**(3):285-289.

3. Alderton WK, Cooper CE, Knowles RG: **Nitric oxide synthases: structure, function and inhibition**. *Biochem J* 2001, **357**(Pt 3):593-615.

4. Dweik RA: **Nitric oxide, hypoxia, and superoxide: the good, the bad, and the ugly!** *Thorax* 2005, **60**(4):265-267.

5. Shaul PW, North AJ, Brannon TS, Ujiie K, Wells LB, Nisen PA, Lowenstein CJ, Snyder SH, Star RA: **Prolonged in vivo hypoxia enhances nitric oxide synthase type I and type III gene expression in adult rat lung**. *Am J Respir Cell Mol Biol* 1995, **13**(2):167-174.

6. Sherman TS, Chen Z, Yuhanna IS, Lau KS, Margraf LR, Shaul PW: **Nitric oxide synthase isoform expression in the developing lung epithelium**. *Am J Physiol* 1999, **276**(2 Pt 1):L383-390.

7. Black SM, Bedolli MA, Martinez S, Bristow JD, Ferriero DM, Soifer SJ: **Expression of neuronal nitric oxide synthase corresponds to regions of selective vulnerability to hypoxia-ischaemia in the developing rat brain**. *Neurobiol Dis* 1995, **2**(3):145-155.

8. Prabhakar NR, Pieramici SF, Premkumar DR, Kumar GK, Kalaria RN: **Activation of nitric oxide synthase gene expression by hypoxia in central and peripheral neurons**. *Brain Res Mol Brain Res* 1996, **43**(1-2):341-346.

9. Huie RE, Padmaja S: **The reaction of NO with superoxide**. *Free Radical Research* 1993, **18**(4):195-199.

10. Pacher P, Beckman JS, Liaudet L: **Nitric oxide and peroxynitrite in health and disease**. *Physiological reviews* 2007, **87**(1):315-424.

11. Mou L, Gates A, Mosser VA, Tobin A, Jackson DA: **Transient hypoxia induces sequestration of M1 and M2 muscarinic acetylcholine receptors**. *J Neurochem* 2006, **96**(2):510-519.

12. Hirota K, Fukuda R, Takabuchi S, Kizaka-Kondoh S, Adachi T, Fukuda K, Semenza GL: **Induction of hypoxia-inducible factor 1 activity by muscarinic acetylcholine receptor signaling**. *J Biol Chem* 2004, **279**(40):41521-41528.

13. Schober DA, Gill MB, Yu H, Gernert DL, Jeffries MW, Ornstein PL, Kato AS, Felder CC, Bredt DS: **Transmembrane AMPA receptor regulatory proteins and cornichon-2 allosterically regulate AMPA receptor antagonists and potentiators**. *J Biol Chem* 2011, **286**(15):13134-13142.

14. Sivakumar V, Foulds WS, Luu CD, Ling EA, Kaur C: **Hypoxia-induced retinal ganglion cell damage through activation of AMPA receptors and the neuroprotective effects of DNQX**. *Exp Eye Res* 2013, **109**:83-97.

15. Storto M, Battaglia G, Gradini R, Bruno V, Nicoletti F, Vairetti M: **Mouse hepatocytes lacking mGlu5 metabotropic glutamate receptors are less sensitive to hypoxic damage**. *Eur J Pharmacol* 2004, **497**(1):25-27.

16. Wang B, Wood IS, Trayhurn P: **Hypoxia induces leptin gene expression and secretion in human preadipocytes: differential effects of hypoxia on adipokine expression by preadipocytes**. *J Endocrinol* 2008, **198**(1):127-134.

17. Bernier NJ, Gorissen M, Flik G: **Differential effects of chronic hypoxia and feed restriction on the expression of leptin and its receptor, food intake regulation and the endocrine stress response in common carp**. *J Exp Biol* 2012, **215**(Pt 13):2273-2282.

18. Huang Q, Viale A, Picard F, Nahon J, Richard D: **Effects of leptin on melanin-concentrating hormone expression in the brain of lean and obese Lep(ob)/Lep(ob) mice**. *Neuroendocrinology* 1999, **69**(3):145-153.
